# Synergies and trade-offs between sanitation and the sustainable development goals

**DOI:** 10.14324/111.444/ucloe.000016

**Published:** 2021-04-27

**Authors:** Priti Parikh, Loan Diep, Pascale Hofmann, Julia Tomei, Luiza C. Campos, Tse-Hui Teh, Yacob Mulugetta, Ben Milligan, Monica Lakhanpaul

**Affiliations:** 1 Engineering for International Development Centre, Bartlett School of Construction Project Management, University College London, 1–19 Torrington Place, London WC1E 7HB, UK; 2 Engineering for International Development Centre, Bartlett School of Construction Project Management, University College London, 1–19 Torrington Place, London WC1E 7HB, UK; 3 Environmental and Sustainable Development, The Bartlett Development Planning Unit, University College London, 34 Tavistock Square, London WC1H 9EZ, UK; 4 Energy, Resources and Development, UCL Institute for Sustainable Resources, 14 Upper Woburn Place, London WC1H 0NN, UK; 5 Environmental Engineering, Centre for Urban Sustainability and Resilience, Civil, Environmental and Geomatic Engineering, University College London, Chadwick Building, London WC1E 6BT, UK; 6 The Bartlett School of Planning, Central House, 14 Upper Woburn Place, London WC1H 0NN, UK; 7 Energy and Development Policy, UCL Department of Science, Technology, Engineering & Public Policy (STEaPP), Shropshire House (4th Fl), 11–20 Caper Street, London WC1E 6JA, UK; 8 Sustainable Development Law and Policy, University of New South Wales, Sydney, NSW 2052, Australia; 9 Integrated Community Child Health, Population, Policy & Practice Department, UCL Great Ormond Street Institute of Child Health, University College London, 30 Guilford Street, London WC1N 1EH, UK; 10 Whittington NHS Trust, Magdala Ave, London N19 5NF, London, UK

**Keywords:** SDG, sanitation, interdisciplinary, cross-sectoral partnerships, synergies, trade-offs, sustainable development, water, the environment

## Abstract

To better leverage opportunities arising out of sustainable and inclusive management of sanitation services there is a need for robust and comprehensive evidence of the wide-ranging benefits that sanitation can deliver. The Sustainable Development Goals (SDGs) provide a comprehensive framework for sustainable development broken down into 169 interconnected Targets which are articulated under 17 Goals. Based on a methodology developed at University College London (UCL), this study identifies linkages between sanitation and the 169 Targets corroborated by published evidence. We show that there are synergies between sanitation and all 17 Goals and 130 (77%) of the Targets, and trade-offs for 28 (17%) of the Targets. We identified 83 Targets (49%) that call for action in the sanitation sector. The results demonstrate the far-reaching benefits that can be unlocked from investment in sanitation, which extend beyond health and spread across sectors. The evidence base for the 17 Goals establishes links that can inform cross-sectoral action, collaborations and investment across governance levels for integrated sanitation solutions. The research provides different stakeholders with a framework that can be applied to context-specific cases and projects. We propose a range of recommendations to policy makers, practitioners and researchers who seek to take this study further to help achieve the SDGs.

## Introduction

In September 2015, the United Nations (UN) adopted the 2030 Agenda for Sustainable Development. Comprising 17 Sustainable Development Goals (SDGs) and 169 Targets, the Agenda proposes a comprehensive global plan of action for ‘people, planet and prosperity’. SDG6 aims to ‘*ensure availability and sustainable management of water and sanitation for all*’ by 2030 ([1], p. 18). It builds on the largely unmet water and sanitation target of the Millennium Development Goals (MDGs) on environmental sustainability; MDG targets failed to support a systemic approach towards sanitation that considered the sanitation chain in its entirety. In 2017, 55% of the world’s population still lacked access to safely managed sanitation, including an estimated two billion who did not have basic access [2]. The severe implications of poor sanitation on morbidity rates, health care costs and productivity losses and inadequate sanitation is estimated to cost the global economy USD 260 billion per year [3]. Our research calls for particular attention to the sanitation dimension of SDG6 and demonstrates that major gains are possible for all goals if universal access to adequate and equitable sanitation is to be achieved.

We argue that the SDGs provide a framework to identify priority areas of investment to maximise impact. An increasing number of studies have adopted such an approach and identified priorities for integrated policies from the analysis of relationships between all SDGs (see e.g. [4–7]). At the sectoral level, assessments have been carried out for links between the SDGs and marine ecology and management [7], energy systems [8–10], climate action [11] and ecosystems [12]. In addition, recent studies have explored linkages between the SDGs and infrastructure systems [13], the SDGs and water [14–16], as well as the water–food–energy nexus among the SDGs [17]. Yet, linkages between sanitation and the SDGs and their potential contributions to public health, the economy and the environment remain under-studied and require evidence-building for practical action.

The Sustainable Sanitation Alliance (SuSanA), an informal network of 11,000 individual members and 353 partner organisations working towards sustainable sanitation solutions, explored the linkages between sanitation and SDGs in order to maximise opportunities to improve access to sanitation [18]. The network highlighted the relevance of sustainable sanitation to meeting the 2030 Agenda by presenting links between sustainable sanitation and all the SDGs and sought to encourage sanitation sector professionals to take action and strive for intersectoral cooperation [18]. Building on the work of SuSanA, this study adds value by presenting a novel and replicable evidence-based methodology that enables a systematic exploration of linkages, disaggregated by actions, synergies and trade-offs, between sanitation systems and the 169 Targets. Revealing these linkages not only highlights the importance of sanitation to other SDGs but can further provide valuable insights into the potential and scope for synergistic efforts towards the 2030 Agenda. The expandable evidence base provided offers a starting point to enhance existing knowledge and demonstrates the value of incorporating sanitation into innovative and integrated approaches and investments.

The aims of this study are twofold: 1) to provide a replicable methodology that establishes linkages with the comprehensive 2030 Agenda and that can be applied in specific contexts to demonstrate the wide-ranging benefits of sanitation that extend across sectors and beyond health; 2) to establish an evidence base of published material to be further expanded as part of efforts to strategically meet the SDGs. Overall, this paper argues that sanitation plays a crucial role in the achievement of the 2030 Agenda and will be key to developing policies and programmes that support sustainable development.

## Methods

A research team from diverse disciplines spanning engineering, urban design and planning, health, social science, political economy, policy and law, worked together from the outset to develop an interdisciplinary approach for knowledge co-production. This approach enables the analysis of complex, interconnected global challenges, providing evidence to support the development of integrated interventions that transcend disciplinary boundaries to develop appropriate approaches and solutions.

The research presented in this paper is based on a definition of safe, inclusive and sustainable sanitation presented in Box 1 which builds upon two concepts. First, SDG Target 6.2, which by 2030 aims to ‘*achieve access to adequate and equitable sanitation and hygiene for all and end open defecation, paying special attention to the needs of women and girls and those in vulnerable situation*’. Second, the sanitation service delivery ladder established by the Joint Monitoring Programme (JMP), which emphasises ‘safely-managed’ sanitation and considers the entire sanitation chain (i.e. capture, containment, emptying, transport, treatment and re-use/disposal) (see also [19–21]) to ensure zero detriment to the environment.

**Box 1. Working definition used for sanitation to ‘achieve adequate and equitable sanitation and hygiene for all’.**

**Achieve access to adequate, equitable and dignified sanitation and hygiene for all, paying attention to:**

– Safely managed facilities and services for handling and disposal of human urine and faeces along the sanitation chain
– Social diversity and inclusivity (including gender, age, disability, religion)
– Capacity-building of local communities
– Menstrual hygiene and baby wash
– Ending open defecation

Based on a review of existing definitions and discussions, the team noted the need to pay particular attention to the diverse needs of sanitation users, including disadvantaged and vulnerable groups (e.g. persons with disabilities), and the importance of menstrual hygiene and baby wash in the provision of sanitation services, which tend to receive less attention [22–24]. In resource-challenged settings, capacity-building of local communities is essential in the provision of sustainable and inclusive sanitation solutions that require an understanding of contextual socio-cultural factors [25]. This study is based on the assumption that only safely managed sustainable sanitation systems are implemented in order to capture the far-reaching benefits that sanitation can deliver and set standards for future infrastructure projects and associated policies.

**Figure 1:**
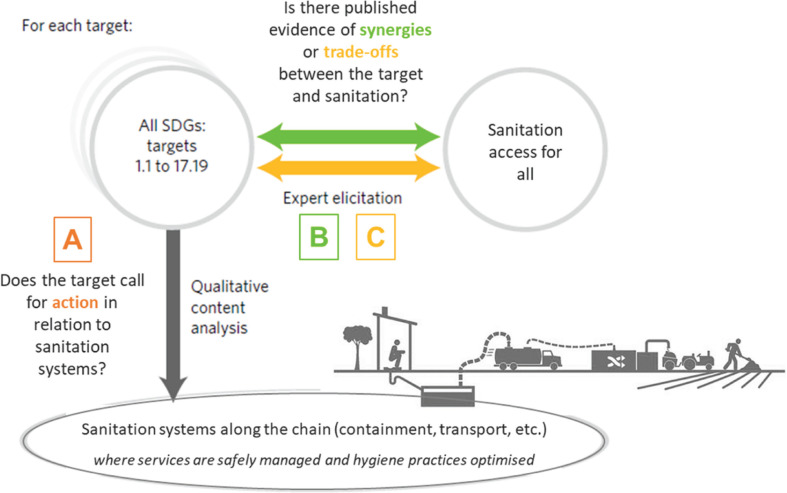
Methodology to assess the interlinkages between sanitation systems and the UN 2030 Agenda (adapted from Fuso Nerini et al. [9]).

In this study, the team adapted the in-house methodology developed at University College London (UCL) [9] for the sanitation sector. Using this structured process, the authors reviewed published evidence to identify linkages between sanitation, as defined above (Box 1), and all the Targets of the 2030 Agenda. The methodology followed a three step-process for each Target, which involved the following questions (see Fig. 1 and Box 2):

**Box 2. Example of identified links (call for action in sanitation, synergies, trade-offs) between sanitation and an SDG target.**

**Target 11.1 – Make cities and human settlements inclusive, safe, resilient and sustainable**

*Target 11.6: By 2030, ensure access for all to adequate, safe and affordable housing and basic services and upgrade slums.*

Two co-authors discussed the call for action, potential synergy and trade-off and used expert knowledge to identify the references included below and in Appendix Table 1.

- **Call for action**: Yes, there is a call for action in sanitation defined as a basic service, in order to provide services of containment, transport, treatment and safe disposal or re-use of excreta, and particularly in low-income areas which remain under-served or un-served (see, e.g. [26]).
- **Synergies**: Yes, access to safe sanitation supports slum upgrading and delivers further benefits from knock-on impacts on housing stock (see, e.g. [27]).
- **Trade-offs**: Yes, if upgrading solutions do not lead to adequate, equitable and dignified sanitation for communities and if there is a mismatch between sanitation interventions and individual aspirations (see, e.g. [28]).

- Does the Target call for action in relation to sanitation, i.e. is an action required in the sanitation sector?
- Is there published evidence of synergies between the pursuit of the Target and achieving access to adequate, equitable and dignified sanitation and hygiene for all?
- Is there published evidence of trade-offs between the pursuit of the Target and achieving access to adequate, equitable and dignified sanitation and hygiene for all?

Using a structured process, the identification of linkages, i.e. call for action, synergies and trade-offs, was initially carried out through a blended approach whereby interdisciplinary pairs used their expertise and knowledge complemented by targeted literature searches to create an interdisciplinary evidence base. Following the method of Fuso Nerini et al. [9], the authors considered at least one piece of published evidence per Target as verification of a synergy or a trade-off. The aim was to test the structured process and the application and replicability of the methodology to demonstrate the importance of sanitation in achieving the 2030 Agenda. In Step A, the authors explored and identified a linkage between sanitation and a Target to assess how improvements in sanitation would affect the Target in question. For Steps B and C, the team looked at reciprocal synergies and trade-offs between sanitation and all SDG targets. In all three steps, the published evidence considered was limited to academic studies and grey literature (e.g. UN reports) published in English. Sources used spanned urban planning, engineering, environment, health, social sciences and policy. The mapping identified existing evidence to indicate the types of synergies and trade-offs that have occurred in different contexts. Box 2 provides an illustrative example of how the methodology was applied for SDG 11.1.

In total, the team scanned over 500 publications. The results are presented in Appendix 1, which references 233 publications to support the identified calls for action, synergies and trade-offs for all 169 Targets. Each Target provides a short explanation of the identified linkages for questions A, B and C. The preliminary results were compiled in the tabulated worksheet (Appendix 1) and scrutinised and validated by all authors, cross-checked in the same pairs, and presented in a workshop with participation from development practitioners engaged in policy, academia and the delivery of sanitation globally.^1^ The tabulated worksheet was then finalised jointly by the authors based on feedback during the workshop. The team was unable to identify published evidence for one Target (15.8 alien species) with potential links with sanitation and was therefore not shown as having a synergistic relationship with sanitation (Appendix 1).

To present the results of our study, all linkages identified have been clustered into four groups referred to as ‘domains’, which were drawn from the frameworks set by Fuso Nerini et al. [9] and Waage et al. [29]. The first group relates to Goals that seek outcomes of ‘individual and collective aspirations of greater welfare and wellbeing’ (SDG1, SDG3, SDG4, SDG5, SDG10, SDG16). The second group, ‘infrastructure services and innovation’, concerns the development of systems of production, distribution and delivery of goods and services (SDG2, SDG6, SDG7, SDG8, SDG9, SDG11, SDG12). The third group refers to ‘the environment and natural resources’ (SDG13, SDG14, SDG15). As in Waage et al. [29], we categorise SDG17 on governance and partnerships separately and thereby frame it as a distinct domain that provides institutional mechanisms which are a prerequisite for the delivery of the targets in groups 1, 2 and 3. As in Fuso Nerini et al. [9], the identification of linkages at Target level (in contrast to Waage et al. [29] who established them at Goal level) showed that similar linkages could exist between sanitation and more than one Target across different Goals.

## Results

**Figure 2:**
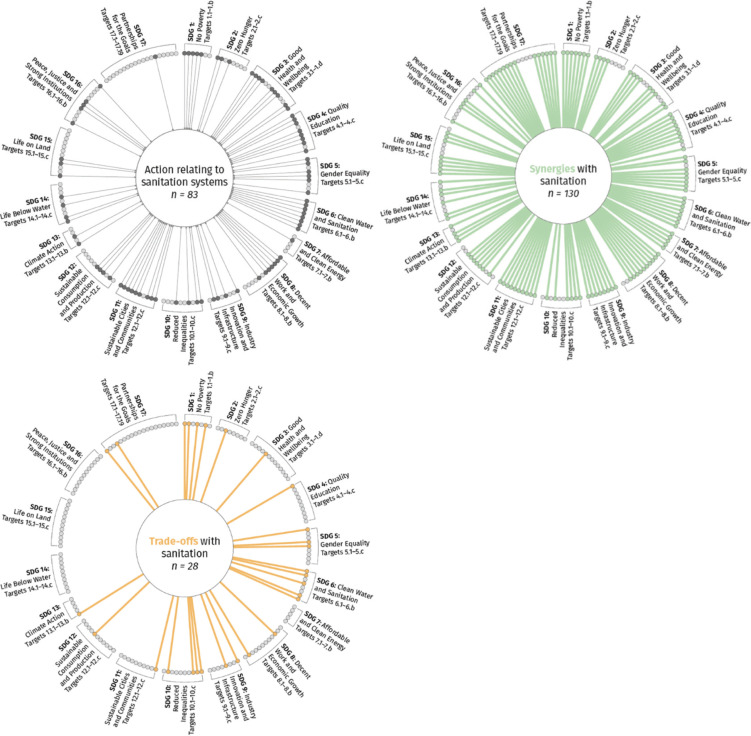
Wheel diagram showing SDGs ‘calling for action (Question A)’, ‘synergies (Question B)’ and ‘trade-off (Question C)’. Diagram adapted from Fuso Nerini et al. [9]. The line represents an evidence call for action, synergy or trade-off and the dot represents each Target.

Our study identified linkages between sanitation and all 17 SDGs highlighting that action on sanitation supports delivery of the 2030 Agenda. Implementation of sustainable sanitation systems would contribute significantly to the achievement of all 17 Goals at multiple levels – individual, household, community, society and environment. As shown in Fig. 2, the assessment found 83 (49%) Targets that required action to be taken concerning sanitation systems. Evidence of synergies and trade-offs were identified for 130 (77%) and 28 (17%) Targets, respectively. The higher number of synergies as compared to trade-offs highlights the wide-ranging benefits of sanitation that can further be explained through our working definition of sustainable and inclusive sanitation, which assumes dignity for all, safe management along the service chain and zero harm to the environment. These are discussed below with reference to a few selected sources of published evidence with the complete results of the assessment reported in Appendix 1 and summarised in Fig. 2. The linkages identified are presented below according to the four domains.

### Individual and collective aspirations of greater welfare and wellbeing

Nine of the 13 Targets of SDG3 (‘Good health and wellbeing’) call for action on sanitation systems. Among all linkages identified, we highlight that sanitation interventions are required to help reduce pathogen transmissions to eliminate maternal and neonatal mortality (Targets 3.1–2); mitigate stress for pregnant women vulnerable to premature deliveries (Target 3.4); and improve hygiene in health care services to promote inclusive access to services (Targets 3.8–9). Several studies confirm that reducing the number of deaths and illnesses caused by air, water and soil pollution depends on the safe disposal of human waste to prevent contamination (Target 3.9) (see for example, [30–32]). There is evidence that lack of access to sanitation is one of the factors leading to diarrhoea and can further reduce the ability to absorb/retain body nutrients leading to malnutrition, which also links to SDG2 (‘Zero Hunger’) [33,34]. It is important to note that the synergistic effects between action on sanitation and faecal–oral disease reduction are well researched, but causal links are yet to be fully corroborated [35,36]. While evidence shows that action in sanitation alone cannot eliminate health issues, this study demonstrates multiple linkages between sanitation and individual and collective wellbeing which go well beyond health.

As sanitation initiatives often fail to target the most marginalised communities, the high-cost burden of inadequate sanitation and poor hygiene these groups subsequently experience make a direct case for investment in sanitation to support SDG1 (‘No Poverty’) and SDG10 (‘Reduced Inequalities’). Safe sanitation can help safeguard health, thereby reducing medical costs, and can deliver additional benefits including time-savings and increased productivity, and can contribute to income generation (Targets 1.1–2) [37,38]. The combination of time saved as a result of safe sanitation access and associated cost savings can have multiplier effects for households where investment enhances sanitation provision at home [30]. Sanitation improvements are further central to promote social, economic and political inclusion, including that of migrants (Targets 10.2, 10.7) [39,40]. However, trade-offs were also identified for three Targets (Targets 1.1–2, 1.a) highlighting how micro-financing structures need to be not only accessible but affordable in order to minimise the financial burden that sanitation investments can represent for households [41–43].

While our study revealed multiple linkages between sanitation and SDG5 (‘Gender Equality’), in practice, gender-inclusive sanitation has yet to be achieved at scale. Action in sanitation is particularly urgent where women and girls bear the brunt of poor infrastructure and services with inadequate access having a knock-on effect on their health, education, disposable income and time-savings (5.1, 5.4) [44]. Sanitation can support SDG5 as well as SDG16 (‘Peace, Justice and Strong Institutions’) regarding the need for safety, for example, through the development of female-friendly toilets, especially where women and girls are exposed to harassment (Targets 5.2, 16.1) [45,46]. There is evidence of the relationship between sanitation and taboos around sexual and reproductive health, and the way lack of access to menstrual hygiene management facilities can affect girls’ school attendance, although further research is needed on this (Targets 5.1–2, 5.6) [47,48]. This link re-emerges in SDG4 (‘Quality Education’), which also presents linkages with sanitation through Water, Sanitation and Hygiene (WASH) and education (at school and elsewhere) (Target 4.3, 4.5) where action on both sides can positively reinforce the other. Several trade-offs were identified in relation to the forms of sanitation and hygiene education delivery, as stigmas (e.g. around menstruation) sometimes perpetuate where action overlooks the need for structural change both with teachers and students (SDG 4.1) [49].

### Infrastructure services and innovation

All Targets in SDG6 (‘Water and Sanitation’) call for action in relation to the delivery of ‘adequate and appropriate’ sanitation and hygiene infrastructure and supporting service systems. There are also possible synergies with all SDG6 Targets, for example, in the way treatment and safe disposal of human faeces and urine safeguards water systems (Targets 6.1, 6.3) [50,51]. Integrated Water Resource Management (IWRM) can benefit sanitation through reclamation of water/use of wastewater (Target 6.4, 6.5) [52], surface water and aquifer conservation (Target 6.6) [53]. However, possible trade-offs within SDG6 occur with the promotion of particular sanitation systems (e.g. flush toilets) that are water-intensive and could impede water security, increase household expenses, and impact on water resources (Targets 6.1–2, 6.4) [50,52]. This emphasises the need to challenge prevalent discourses where improved sanitation is associated with more water-intensive systems.

Sanitation development objectives are closely aligned with the food and energy sectors – SDG2 (‘Zero Hunger’) and SDG7 (‘Affordable and Clean Energy’) – due to the benefits of dealing with human waste as a resource for agricultural production (e.g. Targets 2.1–4 on treated wastewater used for irrigation, sewage sludge for farm productivity) and energy production (e.g. Targets 7.1–2 on human faeces valorisation for biogas production), but such practices are uncommon and typically small-scale and informal and require further evidence-building [54,55]. Synergies between sanitation and all Targets of SDG7 (‘Energy’) for which no trade-offs were identified highlights that investment in sanitation would also benefit other forms of infrastructure that are critical for wellbeing [56,57]. The waste and energy nexus, for example, presents an opportunity to leverage investment across sectors and create multiple social and economic co-benefits [58]. Our definition of sanitation assumes implementation of safe systems covering the entire sanitation value chain, but there will be instances where unsafe disposal or re-use practices may lead to contamination of food and the environment. If such re-use and treatment options are not adequately implemented, this could lead to further contamination of water and soil and present a threat to human health [50,59]. SDG9 (‘Industry, Innovation and Infrastructure’) also recognises the value of resource-efficient systems and emphasises the need for developing and upgrading sanitation infrastructure that enables the valorisation of excreta in closed-loop systems (Targets 9.1, 9.4, 9.a) [60,61].

SDG9 demands investment into innovation, which in the sanitation sector could centre around innovative approaches to existing large-scale sanitation systems to include decentralised schemes. This can include green technologies but also ecological sanitation techniques, such as the development of green infrastructure for wastewater treatment (Targets 9.5, 9.b) [62,63]. Also, on wastewater management, there are four targets under SDG12 (‘Consumption and Production’) calling for change in sanitation infrastructure to re-use waste and thereby reduce pressure on natural resources (Target 12.2, 12.4–6). We identified evidence for different types of synergies to safeguard ecosystems, for example, where irrigation techniques or water-efficient toilets reduce the use of freshwater resources, and where the production of gas and electricity from faecal waste reduces pressure on fossil fuel reserves (Targets 7.2, 12.2, 12.5, 12.8) [61,64,65]. Links between sanitation and SDG13 (‘Climate Action’) include infrastructural change. In parallel to actions seeking to safeguard pressure on water resources, we identify, for example, that integrated sanitation interventions block transmission paths and reduce infection risks in flood-prone areas (Target 13.1) [66,67].

Our study confirms the fundamental role that sanitation has to play in supporting progress towards SDG11 (‘Sustainable Cities and Communities’) and SDG8 (‘Decent Work and Economic Growth’) as a basic service underpinning societal development. Sanitation has a vital role to play in protecting public health in cities as they expand rapidly (Targets 11.1, 11.3) [26–28]. Sanitation supports economic productivity (Targets 8.1–2) and eco-economic decoupling (Target 8.4) [68–71]. Sanitation and SDG8 also link through entrepreneurship, creativity and innovation (Target 9.3) as small and medium enterprises and research and development institutions play pivotal roles in the sanitation sector. However they face various institutional barriers such as access to affordable financing mechanisms and adequate infrastructure [72]. Sanitation action is required to achieve universal ‘decent work’ (Targets 8.5–8), including for sanitation workers – and particularly informal workers involved in faecal sludge management – who are not protected by health and safety measures while they operate in marginalised environments. While efforts are growing to address this challenge they remain ad-hoc and fragmented [73–75].

### Environment and natural resources

Sanitation has a crucial role to play in protecting environmental resources and relates to SDG14 (‘Life Below Water’) and SDG15 (‘Life On Land’) in two important ways: in reducing pollution to conserve ecosystems; and in enhancing ecosystem services through safe re-use of excreta. The latter is important, because the nutrient content of excreta benefits soils and aquatic systems. On reduced pollution, safer or no waste disposal into the environment requires considering the entire sanitation chain rather than adopting a narrow focus on toilets, which is not sufficient to address environmental contamination. We therefore identify the range of actions required where untreated sewage pollutes coastal and marine areas (Targets 14.1–2, 14.5) [76,77], as well as terrestrial and inland freshwater ecosystems (15.1–5) [78]. On the re-use of excreta, published evidence has explored opportunities to restore degraded land and soils (15.2–5) [79–81], as well as to enrich water resources with nutrients (14.2, 14.7) [82,83]. Studies on nature-based solutions have proposed a range of techniques supporting the achievement of objectives in both environmental conservation and sanitation service provision, for example, through wetland conservation or the construction of artificial wetlands (15.1) [84,85].

Synergies were identified for several Targets in relation to ecosystem services and the livelihood opportunities they represent; for example, in aquaculture (Target 14.7) [59,82,86,87], and in sustainable tourism (Target 15.4) to limit the impact of the industry on ecosystems. Furthermore, the water, agricultural and energy sectors are concerned with pollution and waste management (Targets 2.1, 6.1, 6.3, 6.5–6, 7.a) [52,88–90], which is also an important issue tackled by SDG9 (‘Industry, Innovation and Infrastructure’) through Target 9.4 as mentioned in domain 2, and by SDG12 (‘Responsible Consumption and Production’) through Targets 12.2, 12.4–6 as mentioned in domain 3 [60,91,92]. A potential trade-off was identified with Target 15.8 on invasive species as non-native species may be introduced with certain types of sanitation systems (e.g. introduction of alien species through faeces containing seeds), where human waste is applied on soils as a source of nutrients, although no published research was identified to support this.

There is evidence of synergies with integrated climate action reducing environmental contamination from spillage during natural disasters, including that of resilient sanitation infrastructure mentioned in domain 2 (13.1). Building awareness on risks and impacts of climate change on sanitation on-site systems will help the implementation of measures such as the timely emptying of pit latrines and septic tanks in emergency settings to limit environmental impacts (13.3) [66,93]. Recent research has explored the potential of off-site composting of human waste on the reduction of greenhouse gas emissions in the context of container-based sanitation systems in slums, thereby articulating the links between sanitation, climate change, the environment and basic services (13.b, 15.1, 11.1) [94].

### Governance and partnerships for the goals

There are multiple ways through which sanitation relates to the strengthening of institutional mechanisms (SDG17 ‘Partnerships for the Goals’) that can support the achievement of the rest of the SDG Targets. On finance mobilisation and allocation (Targets 17.1–5), there is recognition that the poorest countries receive proportionately less Official Development Assistance (ODA) and that water and sanitation-related ODA is poorly targeted (Target 17.2) [95]. Yet, ODA can sustainably support sanitation interventions on the ground as well as inform policy-making and regulations (Targets 17.2–5) [73,96–100]. Evidence of positive links between sanitation and SDG17 have also been identified in national policies and strategies which capitalise on their international relationships for exchange of sanitation-related information, knowledge, technology and finance (Targets 17.6–9) [101].

Trade-offs may also exist where practices advocated by the international development community set the path for certain practices that do not match local level aspirations or overlook existing local activities [102]. These trade-offs were also identified in other Goals as community needs and/or aspirations are not always reflected in national policy-contexts (e.g. for Targets 6.a, 12.7). Another trade-off relates to the potential of government taxes increasing the financial burden of households and preventing them from investing in sanitation (17.1). Besides, increasing taxes and revenues is not a guarantee that these are used to fund and sustain the expansion of sanitation infrastructures across countries [103]. Some of these challenges are related to the lack of additional finance to pay for sanitation systems that may be costlier than existing ones. Potentially stronger community participation through public–private–civil society partnerships could be a game-changer in the implementation of sanitation systems for which users are willing/able to pay (17.17, 5.5, 6.b, 11.3, 16.7) [104–106]. For example, adapting existing sanitation systems to build climate resilience will require a combination of additional finance and will need deeper participation of the users [107].

At national-level, policy coherence remains an important challenge that requires stronger collaboration between governmental institutions (Target 17.14) [108]. Beyond governments, the formation of strategic multi-stakeholder partnerships will be crucial to achieve the successful planning and implementation of sanitation interventions, including potential beneficiaries (Targets 17.16–17) [109,110]. This is particularly relevant in the context of climate resilience (SDG13) in relation to sanitation services which need to be part of national policies and planning (Target 13.2), and within which community participation is critical (Target 13b). Yet, there is significant uncertainty around climate change impacts, and this means that today’s investments may not result in climate-resilient sanitation systems. Thus, important trade-offs may emerge during the development and adoption of adaptation strategies (Target 13.1). Establishing partnerships will require exploring a range of business models that bring together multiple actors, and building capacity to plan and implement projects collaboratively across levels (Targets 17.4–9). High-quality, timely and reliable data management, including appropriate monitoring and evaluation systems will support the planning, implementation and measurement of progress for sanitation interventions (Targets 17.18–19) [95,111].

## Discussion and recommendations

Our structured review process demonstrates that sanitation action is required to achieve all 17 SDGs. We have identified evidence of synergies between sanitation and 130 Targets out of a total of 169 across the Goals. Synergies exist between sanitation and all Targets that consider inclusivity, social diversity and human wellbeing. Hence, cross-sectoral thinking will result in using resources more effectively, thereby encouraging collaboration and reducing conflict over resources. For example, inclusive sanitation services which embed menstrual hygiene and baby wash management have a direct link with targets in SDG3 and SDG5 that explicitly recognise the diverse needs of girls and women, newborns and children, and vulnerable populations such as the disabled. The rights and dignity of the workforce engaged in sanitation service provision is highlighted through links with SDG9 and SDG10. However, many of the identified trade-offs emphasise possible barriers to inclusive interventions due to conflicting objectives at various scales of action, especially where individual aspirations are overlooked by city, national or global-level strategic agendas. This is as much to do with policy design not taking into account contextual concerns, as it is to do with difficulties encountered with policy implementation. While this study emphasises the need to strengthen governance systems for integrated and cross-sectoral action, this requires further efforts around contextual guidance and documentation which is a gap also identified by Scott et al. [112].

Due to the evidence-based methodology and structured process, our study was able to identify a higher number of linkages between sanitation and the SDG Targets compared to the 2017 SuSanA study referenced in the introduction [18], including less established ones. This concerns, for instance, evidence links between sanitation improvements and SDG10 on reduced inequalities (six Targets), and SDG4 on health, such as the reduction of maternal mortality, the decrease in mortality from non-communicable diseases and the promotion of mental health and wellbeing [113–115].

Wide-ranging and innovative solutions in the sanitation sector are required to achieve resource efficiency, reduction in environmental contamination and improved working conditions in low and middle-income countries, especially for those informally engaged in sanitation service delivery [116]. Meeting the principle of zero harm to the environment would require shifting the focus away from just the provision of toilets to an inclusion of the entire sanitation value chain to include safe sanitation systems. In addition to reducing environmental risk such solutions would also negate associated health risks (diarrhoeal diseases, etc.). Much more than other forms of infrastructure, adaptation and scaling up of sanitation services are highly contingent on and heavily influenced by socio-cultural practices [117]. Hence, adopting participatory approaches and the integration of local population’s knowledge to support decision-making would go some way to develop sanitation solutions which are socio-culturally acceptable.

The synergies and trade-offs documented here are based on a high-level assessment of evidence globally which cuts across geographies, cultures and political contexts. While the global scope of our analysis highlights general implications for sustainable development of action in the sanitation sector it does not offer guidance regarding the degree to which these manifest in specific contexts. Context-specific reviews will be required for different types of sanitation systems to ensure proposed interventions are appropriate and locally relevant. Applying the methodology to concrete case studies will help expand the evidence base established here. Case studies can further help assess the suitability of various technical options for safe sanitation systems to local conditions, acceptance of safe sanitation solutions along the entire value chain to reduce environmental risk, identify complex trade-offs in context, and understand socio-cultural barriers in order to scale-up appropriate solutions. Evidence of the far-reaching impact of sanitation within countries will further provide opportunities to harmonise and leverage in-country investment for sanitation. This would support the development of guidelines and practical tools to enable diverse stakeholders to deliver safe and inclusive sanitation solutions appropriate for local contexts. Context-specific case studies could also include a cross-sectoral evaluation as policy makers often have to compare demands before allocating limited resources.

The following two sections provide recommendations specifically to decision-makers and practitioners involved in sanitation and related sectors regarding how to take the results of this study further. The third section offers suggestions for researchers to replicate this methodology and/or expand the evidence base on linkages between sanitation and the SDGs. The recommendations combine our findings from the study as well as the above-mentioned workshop with development practitioners.

*To governmental institutions and policy makers*: addressing the current institutional and financial fragmentation in sanitation provision will require holistic and integrated policies, underpinned by collaborative and participatory approaches. In most countries, sanitation services are included within public health or water resources ministries where there are multiple and conflicting demands on limited human and financial resources. Depending on the context, addressing the financing gap in sanitation requires either convergence of efforts across ministries or the creation of dedicated cross-sectoral nodal agencies to deliver meaningful sanitation outcomes. Current sanitation investments have been directed to the provision of physical infrastructure in the formal city and less focused on the delivery of services for low-income households and informal settlements [118]. Universalisation of sanitation will require a shift towards co-produced sanitation solutions and inclusive decision-making policies and processes that include the voices of potential users, including marginalised groups [25,119,120]. A range of actors are developing closed-loop systems which provide an opportunity for governmental institutions and policy makers to form inclusive and innovative partnerships.

*To practitioners (including funding institutions, private enterprises, INGOs/NGOs and community-based organisations)*: there is a need to expand evidence on cross-sectoral and multi-level governance collaboration. Practitioners can play an important role in supporting evidence-driven approaches by documenting and disseminating the impact of integrated sanitation interventions. This can be done with support from researchers and used to leverage further funding, in particular for regions and communities currently bypassed by investment. Financing institutions play a fundamental role in supporting the development and scaling-up of innovative solutions for the delivery of adequate, equitable and dignified sanitation interventions through harmonising funding streams to achieve the wide-ranging benefits of sanitation investments evidenced in this study. The private sector, NGOs and community-based organisations will be instrumental in adapting our framework as a participatory monitoring and evaluation tool that can be used to holistically consider impacts of sanitation interventions, socio-cultural factors, and the acceptance of solutions.

*To researchers:* significant research is needed to analyse collaborative investment and intervention models to meet the SDGs. This is key to support practitioners whose resources are often limited to conduct such studies to develop documentation and expand the evidence base. It is crucial to apply the methodology set out in this study in a variety of contexts to build a compendium of case studies with research that reflects realities on the ground and that considers evidence in different languages and goes beyond what has already been published (e.g. verbal testimonies and unpublished data). Similar to Evans and Howard [121], we argue this would support an enriched evidence base and help to substantiate the wide-ranging synergies between sanitation and the SDGs for a variety of socio-political settings. This requires research that embraces the principles of transdisciplinarity and knowledge co-production with active participation from multiple actors including policy actors and end-users to incorporate and embed knowledge in concrete political, geographical and socio-economic settings [122].

## Conclusion

Sanitation as a sector suffers from under-investment of resources. For governments with limited resources, the ability to harmonise efforts across ministries would leverage funding from multiple programmes and initiatives and open the possibility to pitch for funding from sources beyond the traditional sectoral funders. Through the established linkages between sanitation and the 2030 Agenda, this research demonstrates the wide-ranging benefits of sanitation, which extend beyond health across multiple sectors. The identification of synergies locates areas where cross-sectoral thinking will result in using resources more effectively, thereby encouraging collaboration and reducing conflict over resources.

Current sanitation policies and investments bypass marginalised communities and groups, particularly poor women and children and those with disabilities, which in practice has meant that their access to services are deficient at best. A lack of targeted and inclusive policies and actions for those groups impedes progress towards achieving SDG6 and hence all SDGs. In addition, specifically in marginalised areas (informal settlements) knowledge gaps on socially and culturally acceptable technical solutions appropriate for various complex settings result in policy gaps.

This study builds on the need to adopt holistic sanitation systems which consider the entire value chain from safe containment to transport, treatment, disposal and re-use of waste to broker wide reaching benefits and mitigate negative social and environmental impacts. Priority areas in sanitation research for the coming years include understanding and developing re-use and recovery technologies and practices to better integrate a sanitation chain that responds to the water–food–energy nexus. Acceptance of solutions, such as re-use and recovery of human waste, will require acknowledgement of socio-cultural and environmental factors to achieve the direct and tangible impacts that sanitation can have on people and their communities. Shifting the focus away from toilet-centric approaches to a holistic system approach will not only minimise environment risk but would also lead to better health outcomes in terms of reduced morbidity and mortality.

The synergies identified in our study recognise issues where cross-sector thinking would result in more effective use of financial resources, enhanced collaboration and reduced conflict in currently under-served settings. Notably, sanitation has a synergistic link with all or nearly all of the Targets of the Goals for poverty, education, gender, water, energy, industry/infrastructure and cities; this highlights potential synergistic funding opportunities. Given the complexity of the SDGs and the need to integrate multifaceted issues, including climate change, urbanisation, population growth and pressure on environmental resources, experts from different disciplines will need to work together with diverse societal actors to break down traditional silos whilst considering both centralised and decentralised sanitation systems. The methodology presented provides a mechanism that can expand our knowledge base to support the development of more holistic solutions bringing together research, practice and policy actors to create evidence-based policies and practices for integrated resource mobilisation and implementation. It paves the way for transdisciplinary research and practice that can foster inclusive and safe sanitation solutions for all through deeper explorations of context-specific case studies.

This research provides different stakeholders, including policy makers, funders, practitioners and researchers, with a replicable framework. This can advance knowledge and facilitate informed decision-making to enhance funding, planning and implementation of sanitation within transdisciplinary research and practice to achieve the 2030 Agenda. The evidence-base initiated here, while limited in scope and requiring further expansion, can be used as a starting point to leverage and harness investment in sanitation and other sectors to make a difference to the state of sanitation access and address the SDGs more effectively. Most importantly, this is a call for urgent action for everyone to change the status quo to ensure adequate, equitable and inclusive sanitation for all by 2030.

## Data Availability

The datasets generated during and/or analysed during the current study are available from the corresponding author on reasonable request.

## Note

Workshop organised at the Vitol Foundation in September 2019.

## Supporting information

**Appendix 1.** Evidence base from the global review exercise

	Goal or Target in the 2030 Agenda for Sustainable Development	A: Does the target require certain actions in relation to sanitation systems?	B: Synergies between the target and sanitation		C: Trade-offs between the target and sanitation		Reasoning	Sample References
			Identified synergy with sanitation?	Is there evidence for this?	Identified trade-off with sanitation?	Is there evidence for this?		
**Goal 1: End poverty in all its forms everywhere**								
**1.1**	By 2030, eradicate extreme poverty for all people everywhere, currently measured as people living on less than $1.25 a day	**1**	**1**	**1**	**1**	**1**	(A) Eradicating poverty will need to be supported by the provision of access to basic services which includes sanitation and hygiene services (B) Multiple synergies between sanitation and income as poverty is strongly linked to poor human and environmental health which can be due to poor sanitation, while lack of access to adequate sanitation and hygiene is a driver of poverty (C) But also evidence of trade-offs, for example, where improved access requires investment/enhanced expenses that adds financial burden at household level	- Alkire S. How to measure the many dimensions of poverty? In: Organisation for Economic Co-operation and Development, editor. *Development Co-operation Report 2013: Ending Poverty*. OECD Publishing; 2013. - Sah Rb, Khadgi A, Jha N. Study on knowledge and practice regarding sanitation application among the residents of Rangeli Municipality of Morang District, Nepal. *Int J Res Pharm Biosci.* 2017;4:6–12. - Augsburg B, Caeyers B, Oteiza F. *The costs and benefits of investing in a toilet: views from Indian and Nigerian households and their policy implications*. The Institute for Fiscal Studies; 2015.
**1.2**	By 2030, reduce at least by half the proportion of men, women and children of all ages living in poverty in all its dimensions according to national definitions	**1**	**1**	**1**	**1**	**1**	(A) + (B) + (C) as per Target 1.1	- Mehrotra S, Vandermoortele J, Delamonica E. *Basic services for all? Public spending and the social dimensions of poverty*. United Nations Children’s Fund. Florence: Innocenti Research Centre; 2000. - Mara D, Lane J, Scott B, Trouba D. Sanitation and health. *PLoS Med*. 2010;7(11):e1000363. - Hofmann P. *The dialectics of urban water poverty trajectories: policy- driven and everyday practices in Dar es Salaam*. University College London; 2018.
**1.3**	Implement nationally appropriate social protection systems and measures for all, including floors, and by 2030 achieve substantial coverage of the poor and the vulnerable	**1**	**1**	**1**			(A) Target calls for action in sanitation to achieve substantial coverage of the poor and the vulnerable (B) Synergies where social health protection leads to increased access to sanitation and hygiene	- Bachelet M. *Social protection floor for a fair and inclusive globalization*. Geneva: International Labour Office; 2011.
**1.4**	By 2030, ensure that all men and women, in particular the poor and the vulnerable, have equal rights to economic resources, as well as access to basic services, ownership and control over land and other forms of property, inheritance, natural resources, appropriate new technology and financial services, including microfinance	**1**	**1**	**1**	**1**	**1**	(A) Target calls for action in sanitation considered a basic service (B) Synergies by definition (i.e. increased access in sanitation means there is contribution to this target), also evidence that economic resources and other forms of property available to the poor and the vulnerable can lead to increased access to sanitation, including where there are gaps in sanitation investment in tenanted dwellings (C) Several trade-offs including where better sanitation infrastructure increases land and house market and thereby affects access to tenure	- Davis J, White G, Damodaron S, Thorsten R. Improving access to water supply and sanitation in urban India: microfinance for water and sanitation infrastructure development. *Water Sci Technol.* 2008;58:887–91. - Scott P. *Dealing with land tenure and tenancy challenges in water and sanitation services delivery*. London: Water & Sanitation for the Urban Poor; 2013. - Scott P. *Unbundling tenure issues for urban sanitation development.* Loughbourough: Loughborough University; 2011.
**1.5**	By 2030, build the resilience of the poor and those in vulnerable situations and reduce their exposure and vulnerability to climate-related extreme events and other economic, social and environmental shocks and disasters	**1**	**1**	**1**			(A) Target calls for action in sanitation which, as a basic service, directly influences vulnerability to economic, social and environmental shocks and disasters (B) Evidence that improved access to sanitation and health will reduce vulnerability and support resilience-building	- Medland L, Cotton A, Bill B, Foundation MG. *Urban Sanitation Research Programme: consolidated findings*. Loughborough: Water, Engineering and Development Centre (WEDC); 2015.
**1.a**	Ensure significant mobilisation of resources from a variety of sources, including through enhanced development cooperation, in order to provide adequate and predictable means for developing countries, in particular least developed countries, to implement programmes and policies to end poverty in all its dimensions		**1**	**1**	**1**	**1**	(B) Evidence that mobilisation of resources to eradicate poverty has led to improved sanitation (C) But also evidence that mobilisation of certain resources may have led to inequitable or unsustainable sanitation; including due to the way foreign aid is set (e.g. with particular conditionalities) or the way finance is invested	- Wolf S. Does aid improve public service delivery? *Rev World Econ.* 2007;143:650–72. - Trémolet S, Evans B. *Output-based aid for sustainable sanitation*. Washington DC: World Bank; 2010. (OBA Working Paper Series). Report No. 10. - Sinharoy SS, Pittluck R, Clasen T. Review of drivers and barriers of water and sanitation policies for urban informal settlements in low-income and middle-income countries. *Util Policy*. 2019;60:1–8.
**1.b**	Create sound policy frameworks at the national, regional and international levels, based on pro-poor and gender-sensitive development strategies, to support accelerated investment in poverty eradication actions		**1**	**1**			(B) Multiple synergies exist, for example, where the introduction of policy frameworks specifically tackling poverty and/or gender inequalities leads to improved access to sanitation which accelerates poverty eradication itself	- OECD. *Making Poverty Reduction Work: OECD’s Role in Development Partnership*. Paris: Organisation for Economic Co-operation and Development (OECD); 2005.
**Goal 2. End hunger, achieve food security and improved nutrition and promote sustainable agriculture**								
**2.1**	By 2030, end hunger and ensure access by all people, in particular the poor and people in vulnerable situations, including infants, to safe, nutritious and sufficient food all year round	**1**	**1**	**1**			(A) Target calls for action in sanitation for access to safe food, including food that is not contaminated by pathogens (B) Multiple synergies exist, for example, where cleaner water is used for irrigation of crops	- Newell DG, Koopmans M, Verhoef L, Duizer E, Aidara-Kane A, Sprong H, et al. Food-borne diseases – The challenges of 20 years ago still persist while new ones continue to emerge. *Int J Food Microbiol.* 2010;139:S3–S15. - Qadir M, Wichelns D, Raschid-Sally L, McCornick PG, Drechsel P, Bahri A, et al. The challenges of wastewater irrigation in developing countries. *Agric Water Manag.* 2010;97:561–8.
**2.2**	By 2030, end all forms of malnutrition, including achieving, by 2025, the internationally agreed targets on stunting and wasting in children under 5 years of age, and address the nutritional needs of adolescent girls, pregnant and lactating women and older persons	**1**	**1**	**1**			(A) Target calls for action where sanitation issues cause malnutrition, for example, where disease spreading causes diarrhoea which itself causes loss of body nutrients (B) Synergies as per Target 2.1	- Freeman MC, Garn JV, Sclar GD, Boisson S, Medlicott K, Alexander KT, et al. The impact of sanitation on infectious disease and nutritional status: a systematic review and meta-analysis. *Int J Hyg Environ Health.* 2017;220:928–49. - Victora CG, Vaughan JP, Kirkwood BR, Martines JC, Barcelos LB. Risk factors for malnutrition in Brazilian children: the role of social and environmental variables. *Bull World Health Organ*. 1986;64(2):299–309. - Ngure FM, Reid BM, Humphrey JH, Mbuya MN, Pelto G, Stoltzfus RJ. Water, sanitation, and hygiene (WASH), environmental enteropathy, nutrition, and early child development: making the links. *Ann N Y Acad Sci.* 2014;1:118–28.
**2.3**	By 2030, double the agricultural productivity and incomes of small-scale food producers, in particular women, indigenous peoples, family farmers, pastoralists and fishers, including through secure and equal access to land, other productive resources and inputs, knowledge, financial services, markets and opportunities for value addition and non-farm employment		**1**	**1**	**1**	**1**	(B) Synergies exist, for example, where treated sewage is used to increase yield production (e.g. excreta re-use to produce protein feeds for livestock using black soldier flies) and helps nourishing soils (e.g. composted excreta) (C) Trade-offs might exist where conflicts over land-use emerge between agriculture and sanitation-related developments	- Andersson JCM, Zehnder AJB, Rockström J, Yang H. Potential impacts of water harvesting and ecological sanitation on crop yield, evaporation and river flow regimes in the Thukela River basin, South Africa. *Agric Water Manag.* 2011;98:1113–24. - Lydecker M, Drechsel P. Urban agriculture and sanitation services in Accra, Ghana: the overlooked contribution. *Urban Agric Divers Act Benefits City Soc.* 2011;5903:94–103. - Lisansky J. Farming in an urbanizing environment: agricultural land use conflicts and right to farm. *Hum Organ.* 1986;45:363–71.
**2.4**	By 2030, ensure sustainable food production systems and implement resilient agricultural practices that increase productivity and production, that help maintain ecosystems, that strengthen capacity for adaptation to climate change, extreme weather, drought, flooding and other disasters and that progressively improve land and soil quality	**1**	**1**	**1**			(A) Target calls for action in production systems through sustainable practices, including water re-use (B) Synergies as per Target 2.3, and where the use of human excreta is used for cultivating plants that participate in pulling and storing carbon dioxide from the atmosphere	- Kramer S, Preneta N, Kilbride A. Thermophilic composting of human wastes in uncertain urban environments: a case study from Haiti. In: *36th WEDC International Conference: delivering water, sanitation and hygiene services in an uncertain environment*. Nakuru: WEDC, 2013. p. 1–6. - Moya B, Parker A, Sakrabani R, Mesa B. Evaluating the efficacy of fertilisers derived from human excreta in agriculture and their perception in Antananarivo, Madagascar. *Waste Biomass Valo.* 2019;10:941–52.
**2.5**	By 2020, maintain the genetic diversity of seeds, cultivated plants and farmed and domesticated animals and their related wild species, including through soundly managed and diversified seed and plant banks at the national, regional and international levels, and promote access to and fair and equitable sharing of benefits arising from the utilisation of genetic resources and associated traditional knowledge, as internationally agreed							
**2.a**	Increase investment, including through enhanced international cooperation, in rural infrastructure, agricultural research and extension services, technology development and plant and livestock gene banks in order to enhance agricultural productive capacity in developing countries, in particular least developed countries							
**2.b**	Correct and prevent trade restrictions and distortions in world agricultural markets, including through the parallel elimination of all forms of agricultural export subsidies and all export measures with equivalent effect, in accordance with the mandate of the Doha Development Round							
**2.c**	Adopt measures to ensure the proper functioning of food commodity markets and their derivatives and facilitate timely access to market information, including on food reserves, in order to help limit extreme food price volatility							
**Goal 3. Ensure healthy lives and promote wellbeing for all at all ages**								
**3.1**	By 2030, reduce the global maternal mortality ratio to less than 70 per 100,000 live births	**1**	**1**	**1**			(A) Target calls for action in sanitation for women during and post-pregnancy, including for access to adequate and safe toilets and post-birth hygiene (B) Multiple synergies, for example where the elimination of open defecation has led to safer pregnancy in some contexts	- Padhi BK, Baker KK, Dutta A, Cumming O, Freeman MC, Satpathy R, et al. Risk of adverse pregnancy outcomes among women practicing poor sanitation in rural India: a population-based prospective cohort study. *PLoS Med.* 2015;12:1–18. - Baker KK, Story WT, Walser-Kuntz E, Zimmerman MB. Impact of social capital, harassment of women and girls, and water and sanitation access on premature birth and low infant birth weight in India. *PLoS One.* 2018;13:1–18. - Velleman Y, Mason E, Graham W, Benova L, Chopra M, Campbell OMR, et al. From joint thinking to joint action: a call to action on improving water, sanitation, and hygiene for maternal and newborn health. *PLoS Med.* 2014;11:1–9.
**3.2**	By 2030, end preventable deaths of newborns and children under 5 years of age, with all countries aiming to reduce neonatal mortality to at least as low as 12 per 1000 live births and under-5 mortality to at least as low as 25 per 1000 live births	**1**	**1**	**1**			(A) Target calls for action in sanitation for environments that are safe from pathogens (B) Multiple synergies, including in safe sanitation in hospitals, and for the development of handwashing facilities and practices	- Velleman Y, Mason E, Graham W, Benova L, Chopra M, Campbell OMR, et al. From joint thinking to joint action: a call to action on improving water, sanitation, and hygiene for maternal and newborn health. *PLoS Med.* 2014;11:1–9. - Ngure FM, Reid BM, Humphrey JH, Mbuya MN, Pelto G, Stoltzfus RJ. Water, sanitation, and hygiene (WASH), environmental enteropathy, nutrition, and early child development: making the links. *Ann N Y Acad Sci.* 2014;1:118–28.
**3.3**	By 2030, end the epidemics of AIDS, tuberculosis, malaria and neglected tropical diseases and combat hepatitis, water-borne diseases and other communicable diseases	**1**	**1**	**1**			(A) Target calls for action for cleaner water, including through treatment of pathogens (B) Multiple synergies, including in the development of treatment systems for wastewater that contains human waste	- La Rosa G, Fratini M, della Libera S, Iaconelli M, Muscillo M. Emerging and potentially emerging viruses in water environments. *Ann Ist Super Sanita.* 2012;48:397–406. - Mara D. *Domestic wastewater treatment in developing countries*. London: Earthscan; 2003.
**3.4**	By 2030, reduce by one third premature mortality from non-communicable diseases through prevention and treatment and promote mental health and wellbeing	**1**	**1**	**1**			(A) Target calls for action where poor sanitation affects mental health and wellbeing and exposures to risks of non-communicable diseases (NCDs) (B) Evidence shows links between poor sanitation practices and chronic NCDs, for example, through soil-transmitted helminths, also between sanitation and psychological stress, showing synergies between sanitation interventions and NCDs	- Mishra SR, Dhimal M, Bhandari PM, Adhikari B. Sanitation for all: the global opportunity to increase transgenerational health gains and better understand the link between NCDs and NTDs, a scoping review. *Trop Dis Travel Med Vaccines.* 2017;3:1–7. - Marmot M, Friel S, Bell R, Houweling TAJ, Taylor S. Closing the gap in a generation: health equity through action on the social determinants of health. *Lancet.* 2008;372:1661–9. - Hirve S, Lele P, Sundaram N, Chavan U, Weiss M, Steinmann P, et al. Psychosocial stress associated with sanitation practices: experiences of women in a rural community in India. *J Water Sanit Hyg Dev.* 2015;5:115–26. - Padhi BK, Baker KK, Dutta A, Cumming O, Freeman MC, Satpathy R, et al. Risk of adverse pregnancy outcomes among women practicing poor sanitation in rural India: a population-based prospective cohort study. *PLoS Med.* 2015;12:1–18.
**3.5**	Strengthen the prevention and treatment of substance abuse, including narcotic drug abuse and harmful use of alcohol				**1**	**1**	(C) Trade-offs may exist where toilets are used as facilities for drug abuse	- Wolfson-Stofko B, Bennett AS, Elliott L, Curtis R. Drug use in business bathrooms: an exploratory study of manager encounters in New York City. *Int J Drug Policy.* 2017;39:69–77. - Crabtree A, Mercer G, Horan R, Grant S, Tan T, Buxton JA. A qualitative study of the perceived effects of blue lights in washrooms on people who use injection drugs. *Harm Reduct J.* 2013;10:1–8.
**3.6**	By 2020, halve the number of global deaths and injuries from road traffic accidents							
**3.7**	By 2030, ensure universal access to sexual and reproductive health-care services, including for family planning, information and education, and the integration of reproductive health into national strategies and programmes	**1**	**1**	**1**			(A) Target calls for action in sanitation and hygiene in sexual and reproductive health care services (B) Multiple synergies for example, around menstrual hygiene management (MHM)	- IWHC. Integrating sexual and reproductive health in WASH. The International Women’s Health Coalition (IWHC); 2018. - WaterAid, Marie Stopes International Australia. Integrating menstrual health, water, sanitation and hygiene, and sexual and reproductive health in Asia and the Pacific Region. WaterAid and Marie Stopes International Austrialia; 2016.
**3.8**	Achieve universal health coverage, including financial risk protection, access to quality essential health-care services and access to safe, effective, quality and affordable essential medicines and vaccines for all	**1**	**1**	**1**			(A) Target calls for action in sanitation and handwashing services to achieve health coverage for all (B) Synergies since sanitation and hygiene interventions will participate in increasing health coverage	- Lavy V, Strauss J, Thomas D, de Vreyer P. Quality of health care, survival and health outcomes in Ghana. *J Health Econ.* 1996;15:333–57. - Haller L, Hutton G, Bartram J. Estimating the costs and health benefits of water and sanitation improvements at global level. *J Water Health.* 2007;5:467–80.
**3.9**	By 2030, substantially reduce the number of deaths and illnesses from hazardous chemicals and air, water and soil pollution and contamination	**1**	**1**	**1**			(A) Target calls for action in sanitation where there is pollution caused by unsafe human waste disposal and leading to various illnesses and diseases (B) Multiple synergies exist, for example, with treatment, re-use and safe diposal of faeces and urine to reduce water and soil pollution and contamination	- Giusti L. A review of waste management practices and their impact on human health. *Waste Manag.* 2009;29:2227–39. - Montgomery MA, Elimelech M. Water and sanitation in developing countries: including health in the equation – Millions suffer from preventable illnesses and die every year. *Environ Sci Technol.* 2007;41:17–24. - Cairncross S, Valdmanis V. Water supply, sanitation and hygiene promotion. In: *Disease control in developing countries*. Washington DC: The World Bank; 2006. p. 771–92.
**3.a**	Strengthen the implementation of the World Health Organization Framework Convention on Tobacco Control in all countries, as appropriate							
**3.b**	Support the research and development of vaccines and medicines for the communicable and non-communicable diseases that primarily affect developing countries, provide access to affordable essential medicines and vaccines, in accordance with the Doha Declaration on the TRIPS Agreement and Public Health, which affirms the right of developing countries to use to the full the provisions in the Agreement on Trade-related Aspects of Intellectual Property Rights regarding flexibilities to protect public health, and, in particular, provide access to medicines for all							
**3.c**	Substantially increase health financing and the recruitment, development, training and retention of the health workforce in developing countries, especially in least developed countries and Small Island Developing States	**1**	**1**	**1**			(A) Target calls for action in sanitation sector which includes actors who are part of the health workforce (B) Multiple synergies where recruitment, development, training, retention of health workforce improve sanitation and handwashing services	- Rowe G, Frewer LJ. Evaluating public-participation exercises: a research agenda. *Sci Technol Hum Values.* 2004;29:512–57.
**3.d**	Strengthen the capacity of all countries, in particular developing countries, for early warning, risk reduction and management of national and global health risks	**1**	**1**	**1**			(A) Target calls for action in sanitation sector where there are important risks for disease transmission through faecal-oral routes (B) Multiple synergies where transmission is blocked through risk prevention, reduction and management methods before diseases rapidly spread out, for example, cholera, ebola	- Tappero J, Tauxe R. Lessons learned during public health response to cholera epidemic in Haiti and the Dominican Republic. *Emerg Infect. Dis.* 2011;17:2087–93.
**Goal 4. Ensure inclusive and equitable quality education and promote life-long learning opportunities for all**								
**4.1**	By 2030, ensure that all girls and boys complete free, equitable and quality primary and secondary education leading to relevant and effective learning outcomes	**1**	**1**	**1**	**1**	**1**	(A) Ensuring that girls and boys complete education requires action in sanitation, including investment in sanitation facilities in schools (B) Multiple synergies, including where absence of or inadequate sanitation is a cause of school absenteeism; sex-specific toilets encourage attendance among girls; health education in schools contributes to better hygiene and can trigger sanitation and hygiene improvements at home (C) Potential trade-offs may include situations where certain types of health education reproduce sanitation and hygiene issues such as stigmas around menstruation	- Adukia A. Sanitation and education. *Am Econ J Appl Econ.* 2017;9:23–59. - Mahon T, Fernandes M. Menstrual hygiene in South Asia: a neglected issue for WASH (water, sanitation and hygiene) programmes. *Gend Dev.* 2010;18:99–113. - Abrahams N, Mathews S, Ramela P. Intersections of ‘sanitation, sexual coercion and girls’ safety in schools. *Trop Med Int Health.* 2006;11:751–6. - Tellier S, Hyttel M. Menstrual health management in East and Southern Africa: a review paper. In: *Menstrual Health Management Symposium*. Johannesburg: United Nations Population Fund, East and Southern Africa Regional Office; 2018. - Johnston-Robledo I, Chrisler JC. The menstrual mark: menstruation as social stigma. *Sex Roles.* 2013;68:9–18.
**4.2**	By 2030, ensure that all girls and boys have access to quality early childhood development, care and pre-primary education so that they are ready for primary education	**1**	**1**	**1**			(A) Target calls for action in sanitation for ensuring the development of children who are particularly vulnerable to poor sanitation conditions (B) Synergies where higher access to sanitation and hygiene support child growth	- Merchant AT, Jones C, Kiure A, Kupka R, Fitzmaurice G, Herrera MG, et al. Water and sanitation associated with improved child growth. *Eur J Clin Nutr.* 2003;57:1562–8. - Ngure FM, Reid BM, Humphrey JH, Mbuya MN, Pelto G, Stoltzfus RJ. Water, sanitation, and hygiene (WASH), environmental enteropathy, nutrition, and early child development: making the links. *Ann N Y Acad Sci.* 2014;1308:118–28.
**4.3**	By 2030, ensure equal access for all women and men to affordable and quality technical, vocational and tertiary education, including university	**1**	**1**	**1**			(A) Target calls for action in sanitation to support access to tertiary education systems where absence or inadequate sanitation in households and/or in education facilities is a cause of absenteeism (B) Evidence that sanitation that is adequate for different users (including persons with disabilties) leads to the elimination of discrimination and ensures equal access to tertiary education	- Chanana K. Treading the Hallowed Halls women in higher education in India. *Econ Polit Wkly.* 2000;35:1012–22. - Hennegan J, Montgomery P. Do menstrual hygiene management interventions improve education and psychosocial outcomes for women and girls in low and middle income countries? A systematic review. *PLoS One.* 2016;11:1–21.
**4.4**	By 2030, substantially increase the number of youth and adults who have relevant skills, including technical and vocational skills, for employment, decent jobs and entrepreneurship		**1**	**1**			(B) Synergies include job formations benefiting the sanitation sector (e.g masonry); or where the sanitation sector boosts employability of individuals, for example, through provision of trainings; or where sanitation-related formations triggers behaviour change in hygiene practices	- Tracey JB, Cardenas CG. Training effectiveness: an empirical examination of factors outside the training context. *J Hosp Tour Res.* 1996;20:113–23. - Cohen E, Reichel A, Schwartz Z. On the efficacy of an In-House Food Sanitation Training Program: statistical measurements and practical conclusions. *J Hosp Tour Res.* 2001;25:5–16. - Schaub-Jones D. Should we view sanitation as just another business? The crucial role of sanitation entrepreneurship and the need for outside engagement. *Enterp Dev Microfinance.* 2010;21:185–204.
**4.5**	By 2030, eliminate gender disparities in education and ensure equal access to all levels of education and vocational training for the vulnerable, including persons with disabilities, indigenous peoples and children in vulnerable situations	**1**	**1**	**1**			(A) + (B) as per Targets 4.1, 4.3 and 4.4	
**4.6**	By 2030, ensure that all youth and a substantial proportion of adults, both men and women, achieve literacy and numeracy	**1**	**1**	**1**			(A) Target calls for action as sanitation where lack of facilities affects attendance and therefore limits literacy and numeracy achievements (B) Multiple synergies since attendance is linked to adequate and safe sanitation facilities in schools and therefore supports literacy and numeracy achievements; also where achieving literacy and numeracy participates in learning about sanitation and hygiene practices	- Esrey SA, Habicht JP. Maternal literacy modifies the effect of toilets and piped water on infant survival in Malaysia. *Am J Epidemiol.* 1988;127:1079–87. - Rotary. Water. Sanitation. Hygiene. Education. Literacy. A guide to WASH in Schools. Evanston: Rotary International; 2016. - Merchant AT, Jones C, Kiure A, Kupka R, Fitzmaurice G, Herrera MG, et al. Water and sanitation associated with improved child growth. *Eur J Clin Nutr.* 2003;57:1562–8.
**4.7**	By 2030, ensure that all learners acquire the knowledge and skills needed to promote sustainable development, including, among others, through education for sustainable development and sustainable lifestyles, human rights, gender equality, promotion of a culture of peace and non-violence, global citizenship and appreciation of cultural diversity and of culture’s contribution to sustainable development	**1**	**1**	**1**			(A) Education in relation to sanitation and hygiene is required in order to achieve this target (B) Evidence that education in relation to sanitation and hygiene participates in sustainable development, for example in relation to waste disposal; or that knowledge and skills around sustainable development change sanitation and hygiene practices	- Asaolu S, Ofoezie I. The role of health education and sanitation in the control of helminth infections. *Acta Trop.* 2003;86:283–94. - Corcoran E, Nellemann C, Baker E, Bos R, Osborn D, Savelli H. Sick water? The central role of wastewater management in sustainable development: a rapid assessment. Vol. 30, *Mine Water and the Environment*. United Nations Environment Programme, UN-HABITAT, GRID-Arendal; 2010.
**4.a**	Build and upgrade education facilities that are child, disability and gender sensitive and provide safe, non-violent, inclusive and effective learning environments for all	**1**	**1**	**1**			(A) + (B) as per Target 4.1	
**4.b**	By 2020, substantially expand globally the number of scholarships available to developing countries, in particular least developed countries, Small Island Developing States and African countries, for enrolment in higher education, including vocational training and information and communications technology, technical, engineering and scientific programmes, in developed countries and other developing countries							
**4.c**	By 2030, substantially increase the supply of qualified teachers, including through international cooperation for teacher training in developing countries, especially least developed countries and Small Island Developing States	**1**	**1**	**1**			(A) Target calls for action in sanitation where poor conditions negatively affect teachers’ decisions of their teaching location (B) Possible synergies where teachers benefiting from trainings are introduced to health education that will benefit themselves and possibly communities in their home country or that toilets/safe facilities is a pull factor for teachers	- Kayuni H, Tambulasi R. Teacher turnover in Malawi’s Ministry of Education: realities and challenges. *Int Educ J.* 2007;8:89–99. - Adukia A. Sanitation and education. *Am Econ J Appl Econ.* 2017;9:23–59.
**Goal 5. Achieve gender equality and empower all women and girls**								
**5.1**	End all forms of discrimination against all women and girls everywhere	**1**	**1**	**1**	**1**	**1**	(A) Target calls for action in sanitation to end discrimination and recognise women and girls’ special needs (B) Multiple synergies for example, in MHM (C) But also trade-offs if improved access to sanitation perpetuates discrimination towards women, for example, if improvements made at community-level but impedes inspirations at individual level, or if the process does not encourage them to participate	- Sommer M. Putting menstrual hygiene management on to the school water and sanitation agenda. *Waterlines.* 2010;29:268–78. - Mahbub A. Social dynamics of CLTS: inclusion of children, women and the vulnerable. In: *CLTS Conference.* Sussex: IDS; 2008. - Johnston-Robledo I, Chrisler JC. The menstrual mark: menstruation as social stigma. *Sex Roles.* 2013;68:9–18. - Diorio JA, Munro JA. Doing harm in the name of protection: menstruation as a topic for sex education. *Gend Educ.* 2000;12:347–65. - Parikh P, Fu K, Parikh H, McRobie A, George G. Infrastructure provision, gender, and poverty in Indian slums. *World Dev*. 2015;66:468–86.
**5.2**	Eliminate all forms of violence against all women and girls in the public and private spheres, including trafficking and sexual and other types of exploitation	**1**	**1**	**1**			(A) Target calls for action in sanitation to eliminate violence against women and girls, for example in public toilets or in locations of open defecation (B) Synergies, for example, where installation of private toilets or unisex toilets has decreased violence against women, where education around MHM has reduced bullying towards women, or increased personal hygiene habits	- Abrahams N, Mathews S, Ramela P. Intersections of ‘sanitation, sexual coercion and girls’ safety in schools. *Trop Med Int Health.* 2006;11:751–6. - Nath P, Huesco A, Malhotra M, Patel S. *Female-friendly public and community toilets: a guide for planners and decision-makers*. London: UNICEF, WaterAid, WSUP; 2018. - Fisher S. Violence against women and natural disasters: findings from post-tsunami Sri Lanka. *Violence Against Women.* 2010;16:902–18.
**5.3**	Eliminate all harmful practices, such as child, early and forced marriage and female genital mutilation							
**5.4**	Recognise and value unpaid care and domestic work through the provision of public services, infrastructure and social protection policies and the promotion of shared responsibility within the household and the family as nationally appropriate	**1**	**1**	**1**	**1**	**1**	(A) Target calls for action in sanitation to alleviate the burden put on certain women, for example, to take care of household toilets, or baby wash (B) Multiple synergies, for example where the introduction of flush toilets reduces labour for women at home (C) But also risks of trade-offs where introduced system represents more labour to women at home	- O’Keefe M, Lüthi C, Tumwebaze IK, Tobias R. Opportunities and limits to market-driven sanitation services: evidence from urban informal settlements in East Africa. *Environ Urban.* 2015;27:421–40. - Kwiringira J, Atekyereza P, Niwagaba C, Günther I. Gender variations in access, choice to use and cleaning of shared latrines; experiences from Kampala Slums, Uganda. *BMC Public Health.* 2014;14:1–11. - Tumwebaze IK, Mosler HJ. Shared toilet users’ collective cleaning and determinant factors in Kampala slums, Uganda. *BMC Public Health.* 2014;14:1–10. - Parikh P, Fu K, Parikh H, McRobie A, George G. Infrastructure provision, gender, and poverty in Indian slums. *World Dev*. 2015;66:468–86.
**5.5**	Ensure women’s full and effective participation and equal opportunities for leadership at all levels of decision-making in political, economic and public life	**1**	**1**	**1**	**1**	**1**	(A) Call for action in sanitation where poor conditions put barriers to women’s empowerment, for example, where a lack of facilities impedes everyday activities and prevents them from participating to public life (B) Synergies where improved access to sanitation pays attention to women’s needs so they have equal opportunities, or where the sanitation sector gives opportunities to women for them to participate and have access to leadership roles (C) Possible trade-offs if further disproportionate responsibilities are added to women	- Hirai M, Graham JP, Sandberg J. Understanding women’s decision making power and its link to improved household sanitation: the case of Kenya. *J Water Sanit Hyg Dev.* 2016;6:151–60. - UN Women. Gender equality in the 2030 Agenda: gender responsive water and sanitation systems issue brief. New York: UN Women; 2018.
**5.6**	Ensure universal access to sexual and reproductive health and reproductive rights as agreed in accordance with the Programme of Action of the International Conference on Population and Development and the Beijing Platform for Action and the outcome documents of their review conferences	**1**	**1**	**1**			(A) Target calls for action as reproductive healh can be threatened by lack/absence of sanitation access (similar to 3.7) (B) As both the Programme of Action of the International Conference on Population and Development and the Beijing Platform for Action aim to expand access to reproductive health care, there are multiple synergies, for example, where improved access to sanitation services results in reduction of infections or where toilets improve sexual health	- WHO. Women and health: today’s evidence tomorrow’s Agenda. Geneva: WHO; 2009. - Campbell OMR, Benova L, Gon G, Afsana K, Cumming O. Getting the basic rights – the role of water, sanitation and hygiene in maternal and reproductive health: a conceptual framework. *Trop Med Int Health.* 2015;20:252–67. - UN Women. Gender equality in the 2030 Agenda: gender responsive water and sanitation systems issue brief. New York: UN Women; 2018.
**5.a**	Undertake reforms to give women equal rights to economic resources, as well as access to ownership and control over land and other forms of property, financial services, inheritance and natural resources, in accordance with national laws		**1**	**1**			(B) Possible synergies where equal rights to economic resources, access and decision-making by women in relation to land tenure and other forms of property ownership positively change the way sanitation matters are addressed	- Scott P. *Dealing with land tenure and tenancy challenges in water and sanitation services delivery*. London: Water & Sanitation for the Urban Poor; 2013. - Scott P. *Unbundling tenure issues for urban sanitation development*. Loughborough: Loughborough University; 2011.
**5.b**	Enhance the use of enabling technology, in particular information and communications technology, to promote the empowerment of women		**1**	**1**			(B) Synergies where Information and Communications Technology (ICT) are used for women to exchange information and knowledge on sanitation-related matters such as menstruation and menopause	- Bhakta AN. *Opening the doors to the hidden water, sanitation and hygiene needs of women from the onset of the perimenopause in urban Ghana*. Loughborough: Loughborough University; 2019.
**5.c**	Adopt and strengthen sound policies and enforceable legislation for the promotion of gender equality and the empowerment of all women and girls at all levels	**1**	**1**	**1**			(A) Target calls for action in sanitation where policies do not recognise their needs and perpetuate inequality (B) Synergies, for example where policies require better access to toilets by women and girls, or promotes education around menstruation for girls and boys to reduce taboos and harrassment	- Cross P, Coombes Y, editors. *Sanitation and Hygiene in Africa: where do we stand? Analysis from the AfricaSan Conference, Kigali, Rwanda*. London: IWA Publishing; 2014.
**Goal 6. Ensure availability and sustainable management of water and sanitation for all**								
**6.1**	By 2030, achieve universal and equitable access to safe and affordable drinking water for all	**1**	**1**	**1**	**1**	**1**	(A) Target calls for multiple actions in sanitation, for example, for safe water that is free of contaminants (B) Evidence of synergies, for example, in the way treatment and safe disposal of human faeces and urine improves the quality of water, also where ecological toilets reduce demand on water (C) Trade-offs where water-intensive toilets conflicts with saving systems in water management that produce drinking water	- Haq G, Cambridge H. Exploiting the co-benefits of ecological sanitation. *Curr Opin Environ Sustain.* 2012;4:431–5. - Benetto E, Nguyen D, Lohmann T, Schmitt B, Schosseler P. Life cycle assessment of ecological sanitation system for small-scale wastewater treatment. *Sci Total Environ.* 2009;407:1506–16. - Narain S. The flush toilet is ecologically mindless: think about it. *Down to Earth.* 2002;10:1–14. - Hutton G, Chase C. The knowledge base for achieving the sustainable development goal targets on water supply, sanitation and hygiene. *Int J Environ Res Public Health.* 2016;13:1–35.
**6.2**	By 2030, achieve access to adequate and equitable sanitation and hygiene for all and end open defecation, paying special attention to the needs of women and girls and those in vulnerable situations	**1**	**1**	**1**	**1**	**1**	(A) Target calls for action in sanitation by definition (B) Synergies as per definition (C) Trade-offs exist if implemented sanitation systems are not relevant to context or not sustainable (e.g. where newly-built toilets are not matching the target population’s needs, are not maintained over time, are not resilient to climate impacts, or where handwashing practices are not followed), including in emergency contexts where access to sanitation is achieved but making it dignified or long-lasting is challenging (e.g. in zones affected by war), or where external factors intervene (e.g. contamination occurs through contact with animals)	- Zambrano LD, Levy K, Menezes NP, Freeman MC. Human diarrhea infections associated with domestic animal husbandry: a systematic review and meta-analysis. *Trans R Soc Trop Med Hyg.* 2014;108:313–25. - Briceño B, Coville A, Gertler P, Martinez S. Are there synergies from combining hygiene and sanitation promotion campaigns: evidence from a large-scale cluster-randomised trial in rural Tanzania. *PLoS One.* 2017;12:1–19. - Nawab B, Nyborg ILP, Esser KB, Jenssen PD. Cultural preferences in designing ecological sanitation systems in North West Frontier Province, Pakistan. *J Environ Psychol.* 2006;26:236–46.
**6.3**	By 2030, improve water quality by reducing pollution, eliminating dumping and minimising release of hazardous chemicals and materials, halving the proportion of untreated wastewater and substantially increasing recycling and safe reuse globally	**1**	**1**	**1**			(A) Target calls for direct action in sanitation (B) Evidence to support multiple synergies, for example, where treatment and safe re-use of wastewater includes urine and human excreta	- Grant SB, Saphores JD, Feldman DL, Hamilton AJ, Fletcher TD, Cook PLM, et al. Taking the ‘waste’ out of ‘wastewater’ for human water security and ecosystem sustainability. *Science.* 2012;337:681–6. - Narain S. The flush toilet is ecologically mindless: think about it. *Down to Earth.* 2002;10:1–14.
**6.4**	By 2030, substantially increase water-use efficiency across all sectors and ensure sustainable withdrawals and supply of freshwater to address water scarcity and substantially reduce the number of people suffering from water scarcity	**1**	**1**	**1**	**1**	**1**	(A) Target call for direct actions in sanitation (B) Evidence to support multiple synergies, for example with the safe re-use of wastewater as per described in 6.3 (C) Also evidence that some sanitations systems may be water-intensive as per described in 6.1	- Grant SB, Saphores JD, Feldman DL, Hamilton AJ, Fletcher TD, Cook PLM, et al. Taking the ‘waste’ out of ‘wastewater’ for human water security and ecosystem sustainability. *Science.* 2012;337:681–6. - Narain S. The flush toilet is ecologically mindless: think about it. *Down to Earth.* 2002;10:1–14.
**6.5**	By 2030, implement integrated water resources management at all levels, including through transboundary cooperation as appropriate	**1**	**1**	**1**			(A) Target calls for action in sanitation to reduce pollution and discharge in water bodies (B) Evidence that Integrated Water Resources Management (IWRM) principles can benefit sanitation, for example, through re-use of wastewater that includes urine and human excreta and vice versa	- Niemczynowicz J. Urban hydrology and water management – present and future challenges. *Urban Water.* 1999;1:1–14.
**6.6**	By 2020, protect and restore water-related ecosystems, including mountains, forests, wetlands, rivers, aquifers and lakes	**1**	**1**	**1**			(A) Target calls for multiple actions in sanitation, including through reduction of pollution, and the reuse of excreta and urine (B) Evidence that the safe disposal of human excreta and urine is necessary or beneficial to ecosystems, for example, in conserving rivers and aquifers, and in providing nutrients to forests	- Hu GJ, Zhou M, Hou HB, Zhu X, Zhang WH. An ecological floating-bed made from dredged lake sludge for purification of eutrophic water. *Ecol Eng.* 2010;36:1448–58. - Grant SB, Saphores J-D, Feldman DL, Hamilton AJ, Fletcher TD, Cook PLM, et al. Taking the ‘waste’ out of ‘wastewater’ for human water security and ecosystem sustainability. *Science.* 2012;337:681–6. - Foster S, Ait-Kadi M. Integrated Water Resources Management (IWRM): how does groundwater fit in? *Hydrogeol J*. 2012;3:415–8.
**6.a**	By 2030, expand international cooperation and capacity-building support to developing countries in water- and sanitation-related activities and programmes, including water harvesting, desalination, water efficiency, wastewater treatment, recycling and reuse technologies	**1**	**1**	**1**	**1**	**1**	(A) Target calls for action in relation to international cooperation and capacity building in sanitation-related activities and programmes (B) Multiple synergies where international cooperation and capacity-building benefits in-country sanitation systems to increase access (C) Possible trade-offs where gaps exist between different actors favouring different options, for example, where local users reject what supporting countries propose to implement	- Mshandete AM, Parawira W. Biogas technology research in selected sub-saharan African countries – A review. *African J Biotechnol.* 2009;8:116–25. - BORDA. Annual Report 2013-2014. Bremen: BORDA; 2013. - Paterson C, Mara D, Curtis T. Pro-poor sanitation technologies. *Geoforum.* 2007;38:901–7. - Gopalan S, Rajan RS. Has foreign aid been effective in the water supply and sanitation sector? Evidence from panel data. *World Dev.* 2016;85:84–104.
**6.b**	Support and strengthen the participation of local communities in improving water and sanitation management	**1**	**1**	**1**	**1**	**1**	(A) Target calls for action to support and strengthen the participation of local communities in improving sanitation management (B) Multiple synergies, for example in fostering local ownerships of sanitation systems leading to an increased access to locally adequate sanitation services (C) Trade-offs as per Target 6.a or in causing arrangements that are socially disruptive and increase spatial fragmentation and inequalities, for example by adding burden on local communities that initially lack capacity to manage sanitation systems	- Mitlin D, Bartlett S. *Co-production in cities: providing services, empowering communities, changing relationships*. Vol. 38. London: Environment & Urbanization; 2018. - Moretto L, Faldi G, Ranzato M, Rosati FN, Ilito Boozi JP, Teller J. Challenges of water and sanitation service co-production in the global South. *Environ Urban.* 2018;30:425–43. - Nawab B, Nyborg ILP, Esser KB, Jenssen PD. Cultural preferences in designing ecological sanitation systems in North West Frontier Province, Pakistan. *J Environ Psychol.* 2006;26:236–46.
**Goal 7. Ensure access to affordable, reliable, sustainable and modern energy for all**								
**7.1**	By 2030, ensure universal access to affordable, reliable and modern energy services		**1**	**1**			(B) Multiple synergies, including with the development of waste-to-energy systems, for example, with biogas production using human faeces	- Gautam R, Baral S, Herat S. Biogas as a sustainable energy source in Nepal: present status and future challenges. *Renew Sustain Energy Rev.* 2009;13:248–52. - Fangzhou D, Zhenglong L, Shaoqiang Y, Beizhen X, Hong L. Electricity generation directly using human feces wastewater for life support system. *Acta Astronaut.* 2011;68:1537–47.
**7.2**	By 2030, increase substantially the share of renewable energy in the global energy mix	**1**	**1**	**1**			(A) Target calls for action in developing renewable energy which includes the production of biogas with human waste (B) Synergies as per Target 7.1	- Arthur R, Baidoo MF, Antwi E. Biogas as a potential renewable energy source: a Ghanaian case study. *Renew Energy.* 2011;36:1510–6. - Gautam R, Baral S, Herat S. Biogas as a sustainable energy source in Nepal: present status and future challenges. *Renew Sustain Energy Rev.* 2009;13:248–52. - Fangzhou D, Zhenglong L, Shaoqiang Y, Beizhen X, Hong L. Electricity generation directly using human feces wastewater for life support system. *Acta Astronaut.* 2011;68:1537–47.
**7.3**	By 2030, double the global rate of improvement in energy efficiency		**1**	**1**			(B) Various possible synergies to make sanitation technology more efficient, for example, treatment stations	- Abma WR, Driessen W, Haarhuis R, Van Loosdrecht MCM. Upgrading of sewage treatment plant by sustainable and cost-effective separate treatment of industrial wastewater. *Water Sci Technol.* 2010;61:1715–22. - Krzeminski P, Van Der Graaf JHJM, Van Lier JB. Specific energy consumption of membrane bioreactor (MBR) for sewage treatment. *Water Sci Technol.* 2012;65:380–92. - Hanak DP, Kolios AJ, Onabanjo T, Wagland ST, Patchigolla K, Fidalgo B, et al. Conceptual energy and water recovery system for self-sustained nano membrane toilet. *Energy Convers Manag.* 2016;126:352–61.
**7.a**	By 2030, enhance international cooperation to facilitate access to clean energy research and technology, including renewable energy, energy efficiency and advanced and cleaner fossil-fuel technology, and promote investment in energy infrastructure and clean energy technology		**1**	**1**			(B) Synergies include international cooperation having enabled renewable energy development using human excreta and urine for energy production, knowledge exchange and subsequent investment in such type of energy infrastructure and technology	- Mshandete AM, Parawira W. Biogas technology research in selected sub-saharan African countries – a review. *African J Biotechnol.* 2009;8:116–25.
**7.b**	By 2030, expand infrastructure and upgrade technology for supplying modern and sustainable energy services for all in developing countries, in particular least developed countries, Small Island Developing States, and land-locked developing countries, in accordance with their respective programmes of support		**1**	**1**			(B) Synergies as per Target 7.1, with examples of research on domestic biogas production and self-sustained nano-membrane toilet	- Bond T, Templeton MR. History and future of domestic biogas plants in the developing world. *Energy Sustain Dev.* 2011;15:347–54. - Hanak DP, Kolios AJ, Onabanjo T, Wagland ST, Patchigolla K, Fidalgo B, et al. Conceptual energy and water recovery system for self-sustained nano membrane toilet. *Energy Convers Manag.* 2016;126:352–61.
**Goal 8. Promote sustained, inclusive and sustainable economic growth, full and productive employment and decent work for all**								
**8.1**	Sustain per capita economic growth in accordance with national circumstances and, in particular, at least 7% gross domestic product growth per annum in the least developed countries	**1**	**1**	**1**			(A) Target calls for action in sanitation to sustain economic growth through investment in public health and sanitation systems to ensure productive citizens (B) Evidence of multiple synergies, for example, on investment in sanitation and hygiene yields economic returns (e.g. by reducing health costs, return on investments in education)	- Cole MA, Neumayer E. The impact of poor health on total factor productivity. *J Dev Stud.* 2006;42:918–38. - UN-Water. Sanitation is an investment with high economic returns. International year of sanitation. Geneva: UN-Water; 2008. - Van Minh H, Hung NV. Economic aspects of sanitation in developing countries. *Environ Health Insights.* 2011;5:63–70. - Hutton G, Haller L. *Evaluation of the costs and benefits of water and sanitation improvements at the global level*. Geneva: WHO; 2004. - Szreter S. Economic growth, disruption, deprivation, disease, and death: on the importance of the politics of public health for development. *Popul Dev Rev.* 1997;23:693–728. - Hutton G, Chase C. The knowledge base for achieving the sustainable development goal targets on water supply, sanitation and hygiene. *Int J Environ Res Public Health.* 2016;13:1–35.
**8.2**	Achieve higher levels of economic productivity through diversification, technological upgrading and innovation, including through a focus on high-value added and labour-intensive sectors	**1**	**1**	**1**			(A) Target calls for action in the sanitation sector to ensure the diversification and technological upgrade of sanitation systems for the achievement of higher economic productivity (B) Evidence that upgrading of sanitation systems, including through diversification and upgrading of infrastructural systems, and also management and financing systems lead to higher economic productivity; while higher economic productivity can lead to higher investment in sanitation for better access	- Roefs I, Meulman B, Vreeburg JHG, Spiller M. Centralised, decentralised or hybrid sanitation systems? Economic evaluation under urban development uncertainty and phased expansion. *Water Res.* 2017;109:274–86. - O’Keefe M, Lüthi C, Tumwebaze IK, Tobias R. Opportunities and limits to market-driven sanitation services: evidence from urban informal settlements in East Africa. *Environ Urban.* 2015;27:421–40.
**8.3**	Promote development-oriented policies that support productive activities, decent job creation, entrepreneurship, creativity and innovation, and encourage the formalisation and growth of micro-, small- and medium-sized enterprises, including through access to financial services	**1**	**1**	**1**	**1**	**1**	(A) Target calls for policies and finance that support job creation, entrepreneurship and innovation in the sanitation sector (B) Evidence that policies for sanitation services have boosted the sector, for example, through partnerships with small and medium-sized enterprises (SMEs) (C) but also evidence that certain policies may negatively affect workers in the sanitation sector (e.g. impeding informal workers, increased competiveness)	- Scott P, Forte J, Mazeau A. *Barriers and opportunities for sanitation SMEs: a study of the wider market system in Ghana*. London: WSUP; 2017. - Cacouris J. *Recognising and dealing with informal influences in water and sanitation services delivery*. London: WSUP; 2012. - Jeppesen S. Enhancing competitiveness and securing equitable development: can small, micro, and medium-sized enterprises (SMEs) do the trick? *Dev Pract.* 2005;15:463–74.
**8.4**	Improve progressively, through 2030, global resource efficiency in consumption and production and endeavour to decouple economic growth from environmental degradation, in accordance with the 10-year framework of programmes on sustainable consumption and production, with developed countries taking the lead	**1**	**1**	**1**			(A) Action required for decoupling in re-use/recycling/valorisation of human waste (B) Synergies are found where circular economy systems are developed in sanitation and support decoupling	- Simha P, Ganesapillai M. Ecological sanitation and nutrient recovery from human urine: how far have we come? A review. *Sustain Environ Res.* 2017;27:107–16. - Lüthi C, McConville J, Norström A, Panesar A, Ingle R, Saywell D, et al. *Rethinking Sustainable Sanitation for the Urban Domain*. Proceedings of the Water Environment Federation; 2010.
**8.5**	By 2030, achieve full and productive employment and decent work for all women and men, including for young people and persons with disabilities, and equal pay for work of equal value	**1**	**1**	**1**			(A) Target calls for action as sanitation systems are required at the workplace in a way they provide decent jobs for all (B) Evidence that development of the sanitation sector creates employment and supports equal pay, also that improvements in the sanitation sector will increase employability of all women and men, including young people and persons with disabilities	- Chandler TD. Sanitation privatization and sanitation employees’ wages. *J Labor Res.* 1994;15:137–53. - Beagent T. Water and sanitation investments create jobs. 2016. - Ianchovichina E, Estache A, Foucart R, Garsous G, Yepes T. Job creation through infrastructure investment in the Middle East and North Africa. *World Dev.* 2013;45:209–22. - Diagne NA. Health, safety and dignity of sanitation workers: an initial assessment. Washington DC: International Bank for Reconstruction and Development; 2019.
**8.6**	By 2020, substantially reduce the proportion of youth not in employment, education or training	**1**	**1**	**1**			(A) and (B) as for Target 8.5	Same as per Target 8.5
**8.7**	Take immediate and effective measures to eradicate forced labour, end modern slavery and human trafficking and secure the prohibition and elimination of the worst forms of child labour, including recruitment and use of child soldiers, and by 2025 end child labour in all its forms		**1**	**1**			(B) Eradication of forced labour of children involved in sanitation systems (e.g. pit emptying) will participate in making a sector that is equitable and dignified	- Obeng-Odoom F. The future of our cities. *Cities*. 2009;26:49–53. - Grieco M. Living infrastructure: replacing children’s labour as a source of sanitation services in Ghana. *Desalination.* 2009;248:485–93.
**8.8**	Protect labour rights and promote safe and secure working environments for all workers, including migrant workers, in particular women migrants, and those in precarious employment	**1**	**1**	**1**			(A) The target calls for sanitation workers to work in safe and secure working environments (B) Improvements of sanitation systems for workers will contribute to this target, while the achievement of this target will include improvement of sanitation workers’ conditions	- Hirve S, Lele P, Sundaram N, Chavan U, Weiss M, Steinmann P, et al. Psychosocial stress associated with sanitation practices: experiences of women in a rural community in India. *J Water Sanit Hyg Dev.* 2015;5:115–26. - Arbab DM, Weidner BL. Infectious diseases and field water supply and sanitation among migrant farm workers. *Am J Public Health.* 1986;76:694–5. - World Bank, ILO, WaterAid, WHO. Health, safety and dignity of sanitation workers: an initial assessment. Washington DC: World Bank; 2019.
**8.9**	By 2030, devise and implement policies to promote sustainable tourism that creates jobs and promotes local culture and products		**1**	**1**			(B) Evidence of tourism impacting sanitation systems, as well as unsafe sanitation systems impacting the sustainability of tourism, therefore synergies have been identified on both sides for their potential to improve the other	- Nyaupane GP, Thapa B. Perceptions of environmental impacts of tourism: a case study at ACAP, Nepal. *Int J Sustain Dev World Ecol.* 2006;13:51–61. - Parizzi MG, Nunes Menegasse Velasquez L, Uhlein A, Antunes Aranha PR, Martinho Gonçalves J. Environment, tourism and land use planning – Riachinho Basin, Brazil. *Environ Manag Health.* 2001;12:57–66. - Klein RA. Responsible cruise tourism: issues of cruise tourism and sustainability. *J Hosp Tour Manag.* 2011;18:107–16.
**8.10**	Strengthen the capacity of domestic financial institutions to encourage and expand access to banking, insurance and financial services for all		**1**	**1**			(B) Multiple synergies, for example, where capacity-building of financial institutions intervening in the sanitation sector results in increased household access to sanitation facilities and services (e.g. through loans)	- Van Dijk MP, Etajak S, Mwalwega B, Ssempebwa J. Financing sanitation and cost recovery in the slums of Dar es Salaam and Kampala. *Habitat Int.* 2014;43:206–13. - Augsburg B, Rodríguez-Lesmes P. *Sanitation dynamics: toilet acquisition and its economic and social implications*. London: Institute for Fiscal Studies; 2015.
**8.a**	Increase Aid for Trade support for developing countries, in particular least developed countries, including through the Enhanced Integrated Framework for Trade-Related Technical Assistance to Least Developed Countries				**1**	**1**	(C) Aid for Trade excludes the financing of sanitation infrastructure which may limits progress in sanitation	- Hoekman B, Wilson JS. Aid for trade: building on progress today for tomorrow’s future. In: Fardoust S, Kim Y, Sepúlveda C, editors. *Postcrisis growth and development: a Development Agenda for the G-20*. Washington DC: The International Bank for Reconstruction and Development/The World Bank; 2011.
**8.b**	By 2020, develop and operationalise a global strategy for youth employment and implement the Global Jobs Pact of the International Labour Organisation							
**Goal 9. Build resilient infrastructure, promote inclusive and sustainable industrialisation and foster innovation**								

**9.1**	Develop quality, reliable, sustainable and resilient infrastructure, including regional and transborder infrastructure, to support economic development and human well-being, with a focus on affordable and equitable access for all	**1**	**1**	**1**	**1**	**1**	(A) Target calls for change in infrastruture (e.g. toilets, treatment plants) (B) Evidence that sanitation infrastructure supports human wellbeing (C) Possible trade-offs, for example, where focus on infrastructure prevents progress somewhere else along the sanitation chain (e.g. behaviour change, capacity-building, financing). Other potential trade-offs where pressure of ensuring access to sanitation occurs through infrastructure that is not of good quality, reliable, sustainable, or resilient and adds further risks to development and wellbeing	- Beyene H. Sanitation infrastructure sustainability challenges case study: Ethiopia. In: Bongartz P, Vernon N, Fox J, editors. *Sustainable sanitation for all: experiences, challenges and innovations*. Rugby: Practical Action Publishing; 2016. - Howard G, Pedley S, Barrett M, Nalubega M, Johal K. Risk factors contributing to microbiological contamination of shallow groundwater in Kampala, Uganda. *Water Res.* 2003;37:3421–9. - Sahasrabuddhe V. *Making villages open defecation free: issues in institutionalisation of success*. New Delhi; 2015. - Parikh P, Parikh H, Mcrobie A. The role of infrastructure in improving human settlements. *Proc Inst Civ Eng Urban Des Plan*. 2012;166:101–18.
**9.2**	Promote inclusive and sustainable industrialisation and, by 2030, significantly raise industry’s share of employment and gross domestic product, in line with national circumstances, and double its share in least developed countries		**1**	**1**			(B) Synergies include the development of industrial solutions to treat elements (e.g. water, soil) contaminated by faeces and urine; also improved access to sanitation systems by industrial workers will contribute to promoting inclusive and sustainable industrialisation	- Konteh FH. Urban sanitation and health in the developing world: reminiscing the nineteenth century industrial nations. *Health Place*. 2009;15:69–78.
**9.3**	Increase the access of small-scale industrial and other enterprises, in particular in developing countries, to financial services, including affordable credit, and their integration into value chains and markets		**1**	**1**			(B) Synergies, for example, where access to finance by SMEs resulted in their integration in markets, and also led to improvements in access to sanitation services	- Scott P, Forte J, Mazeau A. *Barriers and opportunities for sanitation SMEs: a study of the wider market system in Ghana*. London: Water & Sanitatin for the Urban Poor, 2017. - Tremolet S. *Small-scale finance for water and sanitation*. Stockholm; 2012. - Rahman N. *Adapting and replicating a proven partnership model for urban sanitation: SWEEP in Chittagong*. London: Water & Sanitation for the Urban Poor; 2018.
**9.4**	By 2030, upgrade infrastructure and retrofit industries to make them sustainable, with increased resource-use efficiency and greater adoption of clean and environmentally sound technologies and industrial processes, with all countries taking action in accordance with their respective capabilities	**1**	**1**	**1**	**1**	**1**	(A) Target calls for action to develop industries for more resource efficient and environmentally sound practices (B) Synergies that have been researched include that of human waste used as a combustible in industrial kilns, or used as a fertiliser in the food industry (C) Upgrading infrastructure and retrofitting industries so to make their sanitation systems sustainable might require a lot of investment, including in many Global North contexts	- Gautam R, Baral S, Herat S. Biogas as a sustainable energy source in Nepal: present status and future challenges. *Renew Sustain Energy Rev.* 2009;13:248–52. - Fangzhou D, Zhenglong L, Shaoqiang Y, Beizhen X, Hong L. Electricity generation directly using human feces wastewater for life support system. *Acta Astronaut.* 2011;68:1537–47. - Andersson K, Dickin S, Rosemarin A. Towards ‘sustainable’ sanitation: challenges and opportunities in urban areas. *Sustain.* 2016;8:1289.
**9.5**	Enhance scientific research, upgrade the technological capabilities of industrial sectors in all countries, in particular developing countries, including, by 2030, encouraging innovation and substantially increasing the number of research and development workers per 1 million people and public and private research and development spending		**1**	**1**			(B) Evidence that there are gaps in research on sanitation, for example in research making the case for certain sanitation technologies that are economically feasible and socially acceptable	- Zhou X, Li Z, Zheng T, Yan Y, Li P, Odey EA, et al. Review of global sanitation development. *Environ Int.* 2018;120:246–61.
**9.a**	Facilitate sustainable and resilient infrastructure development in developing countries through enhanced financial, technological and technical support to African countries, least developed countries, landlocked developing countries and Small Island Developing States	**1**	**1**	**1**			(A) The target calls for support to countries in need for sustainable and resilient sanitation infrastructure through financial, technological and technical support (B) Evidence that countries receiving greater aid in sanitation are more likely to reduce health risks including to infants and children	- Botting MJ, Porbeni EO, Joffres MR, Johnston BC, Black RE, Mills EJ. Water and sanitation infrastructure for health: the impact of foreign aid. *Global Health.* 2010;6:12. - Gopalan S, Rajan RS. Has Foreign aid been effective in the water supply and sanitation sector? Evidence from panel data. *World Dev.* 2016;85:84–104.
**9.b**	Support domestic technology development, research and innovation in developing countries, including by ensuring a conducive policy environment for, inter alia, industrial diversification and value addition to commodities	**1**	**1**	**1**			(A) Target calls for support for domestic technological development, research and innovation in sanitation systems (B) Multiple synergies identified, for example, where innovative products and services lead to more equitable, dignified sanitation and hygiene for all	- Murphy HM, McBean EA, Farahbakhsh K. Appropriate technology – a comprehensive approach for water and sanitation in the developing world. *Technol Soc.* 2009;31:158–67. - Diga K. Mobile cell phones and poverty reduction: technology spending patterns and poverty level change among households in Uganda. In: *Workshop on the Role of Mobile Technologies in Fostering Social Development*. São Paulo; 2008. - Parker A. Membrane technology plays key role in waterless hygienic toilet. *Membr Technol.* 2014;12:8.
**9.c**	Significantly increase access to information and communications technology and strive to provide universal and affordable access to the Internet in least developed countries by 2020		**1**	**1**			(B) Evidence of improved sanitation through phone applications used to pay for sanitation services, use of phone apps for early warnings to empty latrines and tanks, manuals consultable on phones and training in humanitarian sector	- GSMA. Loowatt: Digitising the container-based sanitation value chain in Madagascar [Online]. 2017. - Water & Sanitation for the Urban Poor. The PULA app: a customer acquisition and tracking tool for vacuum tanker businesses. London; 2017. - Diga K. Mobile cell phones and poverty reduction: technology spending patterns and poverty level change among households in Uganda. In: *Workshop on the Role of Mobile Technologies in Fostering Social Development*. Sao Paulo; 2008.
**Goal 10. Reduce inequality within and among countries**								
**10.1**	By 2030, progressively achieve and sustain income growth of the bottom 40% of the population at a rate higher than the national average	**1**	**1**	**1**	**1**	**1**	(A) Target calls for action for investment in sanitation to sustain income growth (B) Multiple synergies, for example, where higher access to sanitation reduces costs; where improved sanitation and associated health benefits leads to higher productivity at work; where the sanitation sector creates job opportunities (C) But also evidence of trade-offs, for example, where improved access requires investment that adds financial burden at household level	- Cole MA, Neumayer E. The impact of poor health on total factor productivity. *J Dev Stud.* 2006;42:918–38. - UN-Water. Sanitation is an investment with high economic returns. International year of sanitation. Geneva; 2008. - Whittington D, Lauria DT, Wright AM, Choe K, Hughes JA, Swarna V. Household demand for improved sanitation services in Kumasi, Ghana: a contingent valuation study. *Water Resour Res.* 1993;29:1539–60.
**10.2**	By 2030, empower and promote the social, economic and political inclusion of all, irrespective of age, sex, disability, race, ethnicity, origin, religion or economic or other status	**1**	**1**	**1**	**1**	**1**	(A) Target calls for action in sanitation services for inclusion of all (B) Multiple synergies, including where the introduction of toilets leads to social, economic and political inclusion of individuals (C) Possible trade-offs, for example, where introduction of toilets to households adds layer of exclusion to women (e.g. through decision-making process, or in way it has to be maintained), or where there is discrimination introduced where toilets are gendered/degendered, for example, for transgenders	- DeVries K, Rizo A. Empowerment in action: savings groups improving community water, sanitation, and hygiene services. *Enterp Dev Microfinance.* 2015;26:34–44. - Dankelman I, Muylwijk J, Wendland C, Margriet S. *Making sustainable sanitation work for women and men: integrating a gender perspective into sanitaiton initiatives*. Utrecht; 2009.
**10.3**	Ensure equal opportunity and reduce inequalities of outcome, including by eliminating discriminatory laws, policies and practices and promoting appropriate legislation, policies and action in this regard		**1**	**1**	**1**	**1**	(B) Multiple possible synergie include toilet construction laws, policies and practices that particularly target the poorest, or women and girls (C) Possible trade-offs include the same as for Target 10.2, where attempt to address discrimination to one segment of the populations may increase that of another’s	- Van De Lande L, Ghazi B, Sanghera J. *Eliminating discrimination and inequalities in access to water and sanitation*. Geneve: UN-Water, 2015. - Jeffreys S. The politics of the toilet: a feminist response to the campaign to ‘degender’ a women’s space. *Womens Stud Int Forum.* 2014;45:42–51. - Samar V. The right to privacy and the right to use the bathroom consistent with one’s gender identity. *Duke J Gend Law Policy.* 2016;24:33–59.
**10.4**	Adopt policies, especially fiscal, wage and social protection policies, and progressively achieve greater equality	**1**	**1**	**1**			(A) Target calls for action in sanitation for inclusive policies to meet workers’ needs (B) Synergies where social protection policies ensure minimum sanitation for the most vulnerable, or regulations that specifically target the sanitation labour market and protect sanitation workers from law wages and precarious working conditions	- Koonan S. Background note on prohibition of manual scavenging and protection of the rights of sanitation workers in India. In: *Workshop on Realising the Right to Sanitation – International and Comparative Perspectives*. London: Law, Environment & Development Centre (LEDC); 2013.
**10.5**	Improve the regulation and monitoring of global financial markets and institutions and strengthen the implementation of such regulations							
**10.6**	Ensure enhanced representation and voice for developing countries in decision-making in global international economic and financial institutions in order to deliver more effective, credible, accountable and legitimate institutions							
**10.7**	Facilitate orderly, safe, regular and responsible migration and mobility of people, including through the implementation of planned and well-managed migration policies	**1**	**1**	**1**			(A) Target calls for sanitation systems to be put in place, for example, in context of large population movements (B) Synergies in the achievement of sanitation access, for example, in refugee camps	- Bastable A, Russell L. *Gap analysis in emergency water, sanitation and hygiene promotion*. Humanitarian Innovation Fund. Oxfam GB; 2013. - Jobbins G, Langdown I, Bernard G. *Water and sanitation, migration and the 2030 Agenda for Sustainable Development*. London: Overseas Development Institute; 2018. - Cronin A, Shrestha D, Cornier N, Abdalla F, Ezard N, Aramburu C. A review of water and sanitation provision in refugee camps in association with selected health and nutrition indicators – the need for integrated service provision. *IWA Publ J Water Health.* 2008;6:1–9.
**10.a**	Implement the principle of special and differential treatment for developing countries, in particular least developed countries, in accordance with World Trade Organization agreements							
**10.b**	Encourage official development assistance and financial flows, including foreign direct investment, to States where the need is greatest, in particular least developed countries, African countries, Small Island Developing States and landlocked developing countries, in accordance with their national plans and programmes		**1**	**1**	**1**	**1**	(B) Synergies exist if Official Development Assistance (ODA) is targeted towards achieving access to sanitation (C) Also potential trade-offs if ODA comes with certain conditions which do not match local population’s aspirations (even if conditions are in accordance with national plans), for example, ending open defecation, or promoting sewers versus on-site sanitation	- Cha S, Mankadi PM, Elhag MS, Lee Y, Jin Y. Trends of improved water and sanitation coverage around the globe between 1990 and 2010: Inequality among countries and performance of official development assistance. *Glob Health Action.* 2017;10:1327170. - Gopalan S, Rajan RS. Has foreign aid been effective in the water supply and sanitation sector? Evidence from panel data. *World Dev.* 2016;85:84–104. - Coffey D, Gupta A, Hathi P, Khurana N, Spears D, Srivastav N, et al. Revealed preference for open defecation: evidence from a new survey in Rural North India. *Econ Polit.* 2014;XLIX:43–55.
**10.c**	By 2030, reduce to less than 3% the transaction costs of migrant remittances and eliminate remittance corridors with costs higher than 5%							
**Goal 11. Make cities and human settlements inclusive, safe, resilient and sustainable**								
**11.1**	By 2030, ensure access for all to adequate, safe and affordable housing and basic services and upgrade slums	**1**	**1**	**1**	**1**	**1**	(A) Target calls for action in sanitation (a basic service) by definition in order to provide services of containment, transport, treatment and safe disposal or re-use of excreta, and particularly in low-income areas which remain under-served or un-served (B) Synergies, for example, where access to sanitation contributes to slum upgrading and delivers further benefits from knock-on impacts on housing stock (C) Trade-offs exist, for example if upgrading does not actually lead to adequate, equitable, dignified sanitation, or if community-level sanitation is not meeting individual aspirations	- Bartram J, Charles K, Evans B, O’Hanlon L, Pedley S. Commentary on community-led total sanitation and human rights: should the right to community-wide health be won at the cost of individual rights? *J Water Health.* 2012;10:499–503. - Parikh P, Parikh H, Mcrobie A. The role of infrastructure in improving human settlements. *Proc Inst Civ Eng Urban Des Plan*. 2012;166:101–18. - Dodman D, Leck H, Rusca M, Colenbrander S. African urbanisation and urbanism: implications for risk accumulation and reduction. *Int J Disaster Risk Reduct*. 2017;26:7–15.
**11.2**	By 2030, provide access to safe, affordable, accessible and sustainable transport systems for all, improving road safety, notably by expanding public transport, with special attention to the needs of those in vulnerable situations, women, children, persons with disabilities and older persons	**1**	**1**	**1**			(A) Target calls for action in sanitation to provide facilities in public transport systems that are safe, inclusive and accessible to individuals with respect to their needs, as well as ensuring that trains dispose of human waste safely (B) Synergies exist where transport systems are made more inclusive with sanitation facilities responding to the needs of the most vulnerable, as well as where disposal of waste is done safely	- Raghuram G. *Toilet and Trains*. Ahmedabad: Indian Institute of Management; 2008. - Vegad D, Paruthi S. Sewage disposal system for trains: current system and future prospects. *Int J Emerg Technol.* 2017;8:94–6.
**11.3**	By 2030, enhance inclusive and sustainable urbanisation and capacity for participatory, integrated and sustainable human settlement planning and management in all countries	**1**	**1**	**1**			(A) Target calls for action in sanitation, which as a basic service, will participate in making human settlement planning and management integrated and sustainable (B) Synergies, for example where increase in sanitation access occurs through systems of co-production	- Dodman D, Leck H, Rusca M, Colenbrander S. African urbanisation and urbanism: implications for risk accumulation and reduction. *Int J Disaster Risk Reduct*. 2017;26:7–15. - Shiras T, Cumming O, Brown J, Muneme B, Nala R, Dreibelbis R. Shared sanitation management and the role of social capital: findings from an urban sanitation intervention in Maputo, Mozambique. *Int J Environ Res Public Health.* 2018;15:1–13. - Nance E, Ortolano L. Community participation in urban sanitation: experiences in Northeastern Brazil. *J Plan Educ Res.* 2007;26:284–300.
**11.4**	Strengthen efforts to protect and safeguard the world’s cultural and natural heritage		**1**	**1**			(B) Evidence of tourism impacting sanitation systems, as well as unsafe sanitation systems impacting the sustainability of tourism, therefore synergies have been identified on both sides for their potential to improve the other	- Nyaupane GP, Thapa B. Perceptions of environmental impacts of tourism: a case study at ACAP, Nepal. *Int J Sustain Dev World Ecol.* 2006;13:51–61. - Parizzi MG, Nunes Menegasse Velasquez L, Uhlein A, Antunes Aranha PR, Martinho Gonçalves J. Environment, tourism and land use planning – Riachinho Basin, Brazil. *Environ Manag Health.* 2001;12:57–66. - Klein RA. Responsible cruise tourism: issues of cruise tourism and sustainability. *J Hosp Tour Manag.* 2011;18:107–16.
**11.5**	By 2030, significantly reduce the number of deaths and the number of people affected and substantially decrease the direct economic losses relative to global gross domestic product caused by disasters, including water-related disasters, with a focus on protecting the poor and people in vulnerable situations	**1**	**1**	**1**			(A) Target calls for action in sanitation to reduce the impacts of disasters, for example where pathogen risks are higher, where disasters affect sanitation facilities and services, or where they lead to populations’ displacement which require the creation of emergency services (B) Multiple synergies, for example, in building resilient sanitation infrastructure, in triggering early warnings to empty latrines, and, from a social learning perspective, in developing management systems that are less exposed to risks	- Ersel M. Water and sanitation standards in humanitarian action. *Turkish J Emerg Med*. 2015;15:27–33. - Sherpa AM, Koottatep T, Zurbrügg C, Cissé G. Vulnerability and adaptability of sanitation systems to climate change. *J Water Clim Change*. 2014;5:487–95. - Johannessen Å, Rosemarin A, Thomalla F, Gerger Swartling Å, Axel Stenström T, Vulturius G. Strategies for building resilience to hazards in water, sanitation and hygiene (WASH) systems: the role of public private partnerships. *Int J Disaster Risk Reduct.* 2014;10:102–15.
**11.6**	By 2030, reduce the adverse per capita environmental impact of cities, including by paying special attention to air quality and municipal and other waste management	**1**	**1**	**1**			(A) Target calls for action in human waste disposal (B) Synergies exist around the development of disposal systems that reduce the ecological impacts of cities, for example by reducing the use of chemicals for treatment	- Bartram J, Charles K, Evans B, O’Hanlon L, Pedley S. Commentary on community-led total sanitation and human rights: should the right to community-wide health be won at the cost of individual rights? *J Water Health*. 2012;10:499–503. - Cronin AA, Hoadley AW, Gibson J, Breslin N, Komou FK, Haldin L, et al. Urbanisation effects on groundwater chemical quality: findings focusing on the nitrate problem from 2 African cities reliant on on-site sanitation. *J Water Health.* 2007;5:441–54. - Winker M, Vinnerås B, Muskolus A, Arnold U, Clemens J. Fertiliser products from new sanitation systems: their potential values and risks. *Bioresour Technol.* 2009;100:4090–6. - Spångberg J, Tidåker P, Jönsson H. Environmental impact of recycling nutrients in human excreta to agriculture compared with enhanced wastewater treatment. *Sci Total Environ.* 2014;493:209–19.
**11.7**	By 2030, provide universal access to safe, inclusive and accessible, green and public spaces, in particular for women and children, older persons and persons with disabilities	**1**	**1**	**1**			(A) Target calls for action in sanitation to provide facilities in green and public spaces that are safe, inclusive and accessible to individuals with respect to their needs (B) Multiple synergies including in the way green and public spaces are developed so they provide nature-based water treatment systems	- Greed C. Public toilets: the need for compulsory provision. *Proc Inst Civ Eng Munic Eng.* 2004;157:77–85. - Davar N. *Towards an integrated infrastructure: using architecture to celebrate a Canadian National Park Town*. Dalhousie University; 2013. - Qian YL, Mecham B. Long-term effects of recycled wastewater irrigation on soil chemical properties on golf course fairways. *Agron J.* 2005;97:717–21.
**11.a**	Support positive economic, social and environmental links between urban, peri-urban and rural areas by strengthening national and regional development planning	**1**	**1**	**1**			(A) Target calls for action to provide sanitation of part of urban and regional plans (B) Multiple synergies as national and regional development planning may include improvements in sanitation access	- Ramôa A, Lüthi C, McConville J, Matos J. Urban sanitation technology decision-making in developing countries: a critical analysis of process guides. *Int J Urban Sustain Dev.* 2016;8:191–209.
**11.b**	By 2020, substantially increase the number of cities and human settlements adopting and implementing integrated policies and plans towards inclusion, resource efficiency, mitigation and adaptation to climate change, resilience to disasters, and develop and implement, in line with the Sendai Framework for Disaster Risk Reduction 2015–2030, holistic disaster risk management at all levels	**1**	**1**	**1**			(A) Target calls for action in sanitation to implement policies and plans for inclusion, resource efficiency and resilience-building (B) Multiple synergies as actions in sanitation can contribute to rendering cities and human settlements more inclusive if special needs are addressed, resource efficient (e.g. with less energy- and water-intensive sanitation technologies) and resilient to the impacts of disasters (e.g. with climate-proof sanitation technologies)	- The World Bank. Climate change, disaster risk, and the urban poor: cities building resilience for a changing world. In: Baker JL, editor. Washington DC: The World Bank; 2012.
**11.c**	Support least developed countries, including through financial and technical assistance, in building sustainable and resilient buildings utilising local materials	**1**	**1**	**1**			(A) Target calls for action in sanitation as sustainable and resilient buildings require sanitation interventions (B) Synergies include interventions to make buildings sustainable and resilient through sanitation interventions	- Melchert L. The Dutch sustainable building policy: a model for developing countries? *Build Environ.* 2007;42:893–901.
**Goal 12. Ensure sustainable consumption and production patterns**								
**12.1**	Implement the 10-year framework of programmes on sustainable consumption and production, all countries taking action, with developed countries taking the lead, taking into account the development and capabilities of developing countries							
**12.2**	By 2030, achieve the sustainable management and efficient use of natural resources	**1**	**1**	**1**			(A) Target calls for action in sanitation, for enhancement of ecosystems but also for conservation (B) Multiple synergies exist, for example, where water saving toilets reduce the use of water resources, production of gas and electricity from faecal waste to reduce pressure on fossil fuel reserves	- Afifi S, Alnahhal S, Abdelall S. Developing an integrated sustainable sanitation system for urban areas: Gaza Strip case study. *Procedia CIRP.* 2015;26:767–74. - Cordell D, Rosemarin A, Schröder JJ, Smit AL. Towards global phosphorus security: a systems framework for phosphorus recovery and reuse options. *Chemosphere.* 2011;84:747–58. - Mshandete AM, Parawira W. Biogas technology research in selected sub-saharan African countries – a review. *African J Biotechnol.* 2009;8:116–25.
**12.3**	By 2030, halve per capita global food waste at the retail and consumer levels and reduce food losses along production and supply chains, including post-harvest losses							
**12.4**	By 2020, achieve the environmentally sound management of chemicals and all wastes throughout their life cycle, in accordance with agreed international frameworks, and significantly reduce their release to air, water and soil in order to minimise their adverse impacts on human health and the environment	**1**	**1**	**1**			(A) Target calls for action towards waste management, which includes human excreta and urine (B) Multiple synergies as per Target 12.2	- Asano T, Levine AD. Wastewater reclamation, recycling and reuse: past, present, and future. *Water Sci Technol.* 1996;33:1–14.
**12.5**	By 2030, substantially reduce waste generation through prevention, reduction, recycling and reuse	**1**	**1**	**1**			(A) Target calls for action in waste management which includes human excreta and urine (B) Multiple synergies, including where human waste is treated through natural systems (as in 12.2) and re-used for energy production (as in 7.1) or for irrigation (as in 2.3)	- Fangzhou D, Zhenglong L, Shaoqiang Y, Beizhen X, Hong L. Electricity generation directly using human feces wastewater for life support system. *Acta Astronaut.* 2011;68:1537–47. - Andersson JCM, Zehnder AJB, Rockström J, Yang H. Potential impacts of water harvesting and ecological sanitation on crop yield, evaporation and river flow regimes in the Thukela River basin, South Africa. *Agric Water Manag.* 2011;98:1113–24. - Mshandete AM, Parawira W. Biogas technology research in selected sub-saharan African countries – a review. *African J Biotechnol.* 2009;8:116–25.
**12.6**	Encourage companies, especially large and transnational companies, to adopt sustainable practices and to integrate sustainability information into their reporting cycle	**1**	**1**	**1**			(A) Target calls for action by companies in their management of human waste (B) Synergies where human waste is internally valorised in a closed-loop system (e.g. energy production, irrigation)	- Gensch R. Agriculture and sanitation. *Urban Agric.* 2008;20:38–40. - Toilet Board Coalition. The circular sanitation economy: new pathways to commercial and societal benefits. Geneva: Toilet Board Coalition; 2017.
**12.7**	Promote public procurement practices that are sustainable, in accordance with national policies and priorities		**1**	**1**	**1**	**1**	(B) Synergies exist where procurement practices encourage the use of sustainable sanitation goods and services (C) Potential trade-offs include where these procurement practices set the path for certain practices that are not matching aspirations at local level, or overlook existing local activities	- Econ. Assessing the impact of public sector procurement on competition. London: Econ; 2004. - Khan S. *Swachh Bharat Mission (urban): needs vs planning*. New Delhi; 2018. - Rheinländer T, Samuelsen H, Dalsgaard A, Konradsen F. Hygiene and sanitation among ethnic minorities in Northern Vietnam: does government promotion match community priorities? *Soc Sci Med.* 2010;71:994–1001.
**12.8**	By 2030, ensure that people everywhere have the relevant information and awareness for sustainable development and lifestyles in harmony with nature		**1**	**1**			(B) Synergies where knowedge of sutainable sanitation sytems expands and reduces pressure on natural systems	- Lamichhane KM, Babcock RW. Survey of attitudes and perceptions of urine-diverting toilets and human waste recycling in Hawaii. *Sci Total Environ.* 2013;443:749–56. - Euler H, Aibeo P. Application of ecosan principles through public private partnership projects – prospects and limitations. In: Wener C, et al., editor. 2nd International Symposium. Eschborn: IWA and GTZ; 2004.
**12.a**	Support developing countries to strengthen their scientific and technological capacity to move towards more sustainable patterns of consumption and production		**1**	**1**			(B) Synergies where sustainable sanitation systems (e.g. ecological that do not add pressure on natural resources) are developed as a result of strengthened scientific and technological capacity	- Katukiza AY, Ronteltap M, Niwagaba CB, Foppen JWA, Kansiime F, Lens PNL. Sustainable sanitation technology options for urban slums. *Biotechnol Adv.* 2012;30:964–78. - Kengne IM, Dodane P-H, Akoa A, Koné D. Vertical-flow constructed wetlands as sustainable sanitation approach for faecal sludge dewatering in developing countries. *Desalination.* 2009;248:291–7.
**12.b**	Develop and implement tools to monitor sustainable development impacts for sustainable tourism that creates jobs and promotes local culture and products	**0**			**0**		
**12.c**	Rationalise inefficient fossil-fuel subsidies that encourage wasteful consumption by removing market distortions, in accordance with national circumstances, including by restructuring taxation and phasing out those harmful subsidies where they exist, to reflect their environmental impacts, taking fully into account the specific needs and conditions of developing countries and minimising the possible adverse impacts on their development in a manner that protects the poor and the affected communities							
**Goal 13. Take urgent action to combat climate change and its impacts**								
**13.1**	Strengthen resilience and adaptive capacity to climate-related hazards and natural disasters in all countries	**1**	**1**	**1**	**1**	**1**	(A) Target calls for action as climate change impacts increasingly require adaptive sanitation systems (e.g. climate-proof infrastructure where there are sanitation-related contamination risks during floods or droughts, management systems that can trigger early warnings) (B) Evidence of multiple synergies, for example where resilient sanitation infrastructure blocks transmission paths; or where emptying systems are designed to limits risks of contamination during flood events (C) Possible trade-offs where sanitation actions are no longer adapted to climate change impacts in the short- and/or long-term future	- Lipa M. Health and Sanitation Aspects of Flood Management. Integrated flood management tools series. 2015. - Howard G, Calow R, Macdonald A, Bartram J. Climate change and water and sanitation: likely impacts and emerging trends for action. *Annu Rev Environ Resour.* 2016;41:253–76. - Sherpa AM, Koottatep T, Zurbrügg C, Cissé G. Vulnerability and adaptability of sanitation systems to climate change. *J Water Clim Change.* 2014;5:487–95. - Kohlitz JP, Chong J, Willetts J. Climate change vulnerability and resilience of water, sanitation, and hygiene services: a theoretical perspective. *J Water Sanit Hyg Dev.* 2017;7:181–95. - Oates N, Ross I, Calow R, Carter R, Doczi J. Adaptation to climate *change in water, sanitation and hygiene: assessing risks and appraising options in Africa*. London: ODI; 2014.
**13.2**	Integrate climate change measures into national policies, strategies and planning		**1**	**1**			(B) Multiple synergies where climate-related national measures lead to the strengthening of sanitation sytems to increase resilience to impacts, or where changes made to sanitation systems lead to increased resilience to climate change	- Oates N, Ross I, Calow R, Carter R, Doczi J. *Adaptation to climate change in water, sanitation and hygiene: assessing risks and appraising options in Africa.* London: ODI; 2014.
**13.3**	Improve education, awareness-raising and human and institutional capacity on climate change mitigation, adaptation, impact reduction and early warning	**1**	**1**	**1**			(A) Target calls for action in sanitation sector to better mitigate, adapt to and reduce climate change and its impacts (B) Multiple synergies, including where increased understanding of risks and impacts can improve response for example early warnings to empty on-site sanitation systems	- Keim ME. Building Human resilience. *Am J Prev Med.* 2008;35:508–16. - Oates N, Ross I, Calow R, Carter R, Doczi J. *Adaptation to climate change in water, sanitation and hygiene: assessing risks and appraising options in Africa*. London: ODI; 2014. - Howard G, Calow R, Macdonald A, Bartram J. Climate change and water and sanitation: likely impacts and emerging trends for action. *Annu Rev Environ Resour.* 2016;41:253–76. - Fewster E. Resilient WASH systems in flood-prone areas; techniques to improve the resilience of community WASH systems in flood-prone areas. 2012.
**13.a**	Implement the commitment undertaken by developed-country parties to the United Nations Framework Convention on Climate Change to a goal of mobilising jointly $100 billion annually by 2020 from all sources to address the needs of developing countries in the context of meaningful mitigation actions and transparency on implementation and fully operationalise the Green Climate Fund through its capitalisation as soon as possible		**`**					
**13.b**	Promote mechanisms for raising capacity for effective climate change-related planning and management in least developed countries and Small Island Developing States, including focusing on women, youth and local and marginalised communities		**1**	**1**			(B) Synergies where mechanisms such as community participation or partnerships in developing countries enhance sanitation planning and management to adapt to climate change impacts	- Sherpa AM, Koottatep T, Zurbrügg C, Cissé G. Vulnerability and adaptability of sanitation systems to climate change. *J Water Clim Change.* 2014;5:487–95. - Kohlitz JP, Chong J, Willetts J. Climate change vulnerability and resilience of water, sanitation, and hygiene services: a theoretical perspective. *J Water Sanit Hyg Dev.* 2017;7:181–95. - McNicol G, Jeliazovski J, François JJ, Kramer S, Ryals R. Climate change mitigation potential in sanitation via off-site composting of human waste. *Nat Clim Change*. 2020;6:545–9.
**Goal 14. Conserve and sustainably use the oceans, seas and marine resources for sustainable development**								
**14.1**	By 2025, prevent and significantly reduce marine pollution of all kinds, in particular from land-based activities, including marine debris and nutrient pollution	**1**	**1**	**1**			(A) Target requires action towards reduced marine pollution, including from unsafe waste disposal (B) Evidence that safe treatment/re-use/disposal will reduce pollution	- Baum R, Luh J, Bartram J. Sanitation: a global estimate of sewerage connections without treatment and the resulting impact on MDG progress. *Environ Sci Technol.* 2013;47:1994–2000. - Shuval H. Estimating the global burden of thalassogenic diseases: human infectious diseases caused by wastewater pollution of the marine environment. *J Water Health.* 2003;1:53–64.
**14.2**	By 2020, sustainably manage and protect marine and coastal ecosystems to avoid significant adverse impacts, including by strengthening their resilience, and take action for their restoration in order to achieve healthy and productive oceans	**1**	**1**	**1**			(A) Target calls for action as sanitation systems need to be non-polluting (B) Research has looked into the link between sanitation and marine and coastal ecosystems, which can benefit from nutrient enrichment, or better protection from pollution if sanitation treatment/re-use/disposal is safe	- Aronson RB, Thatje S, Mcclintock JB, Hughes KA. Anthropogenic impacts on marine ecosystems in Antarctica. *Ann N Y Acad Sci.* 2011;1223:82–107. - Morris RM, Nunn BL, Frazar C, Goodlett DR, Ting YS, Rocap G. Comparative metaproteomics reveals ocean-scale shifts in microbial nutrient utilization and energy transduction. *ISME J.* 2010;4:673–85.
**14.3**	Minimise and address the impacts of ocean acidification, including through enhanced scientific cooperation at all levels							
**14.4**	By 2020, effectively regulate harvesting and end overfishing, illegal, unreported and unregulated fishing and destructive fishing practices and implement science-based management plans, in order to restore fish stocks in the shortest time feasible, at least to levels that can produce maximum sustainable yield as determined by their biological characteristics							
**14.5**	By 2020, conserve at least 10% of coastal and marine areas, consistent with national and international law and based on the best available scientific information	**1**	**1**	**1**			(A) Target calls for action in the protection of coastal and marine areas that are affected by contaminated sewage effluents (B) Synergies as sustainable sanitation systems reduce marine pollution which reinforces the area-based conservation of marine areas	Same as for 14.1
**14.6**	By 2020, prohibit certain forms of fisheries subsidies which contribute to overcapacity and overfishing, eliminate subsidies that contribute to illegal, unreported and unregulated fishing and refrain from introducing new such subsidies, recognising that appropriate and effective special and differential treatment for developing and least developed countries should be an integral part of the World Trade Organization fisheries subsidies negotiation							
**14.7**	By 2030, increase the economic benefits to Small Island Developing States and least developed countries from the sustainable use of marine resources, including through sustainable management of fisheries, aquaculture and tourism	**1**	**1**	**1**			(A) Call for action in societies whose livelihood directly depends on wastewater aquaculture (B) Evidence that fisheries relying on wastewater can constitute a sustainable form of aquaculture and plays a part in poor populations’ livelihood	- Bunting SW. Wastewater aquaculture: perpetuating vulnerability or opportunity to enhance poor livelihoods? *Worlds Poult Sci J.* 2004;1:51–75. - Kumar B, Kumar KS, Priya M, Mukhopadhyay D, Shah R. Distribution, partitioning, bioaccumulation of trace elements in water, sediment and fish from sewage fed fish ponds in eastern Kolkata, India. *Toxicol Environ Chem.* 2010;92:243–60. - Edwards P. Key issues in the safe use of wastewater and excreta in aquaculture. Vol. 3, Third edition of the Guidelines for the Safe Use of Wastewater, Excreta and Greywater in Agriculture and Aquaculture. 2010. - Edwards PET. *Reuse of human wastes in aquaculture: a technical review* [Online]. Washington DC; 1992.
**14.a**	Increase scientific knowledge, develop research capacity and transfer marine technology, taking into account the Intergovernmental Oceanographic Commission Criteria and Guidelines on the Transfer of Marine Technology, in order to improve ocean health and to enhance the contribution of marine biodiversity to the development of developing countries, in particular Small Island Developing States and least developed countries		**1**	**1**			(B) Synergies identified, for example, where advance in research methods and knowledge in sanitation supports the understanding of systems that can improve ocean health	- Nichols PD, Leeming R, Rayner MS, Latham V. Use of capillary gas chromatography for measuring fecal-derived sterols application to stormwater, the sea-surface microlayer, beach greases, regional studies, and distinguishing algal blooms and human and non-human sources of sewage pollution. *J Chromatogr A.* 1996;733:497–509. - Ziajahromi S, Neale PA, Leusch FDL. Wastewater treatment plant effluent as a source of microplastics: review of the fate, chemical interactions and potential risks to aquatic organisms. *Water Sci Technol.* 2016;74:2253–69.
**14.b**	Provide access for small-scale artisanal fishers to marine resources and markets						
**14.c**	Enhance the conservation and sustainable use of oceans and their resources by implementing international law as reflected in UNCLOS, which provides the legal framework for the conservation and sustainable use of oceans and their resources, as recalled in paragraph 158 of The Future We Want		**1**	**1**			(B) Synergies identified, for example, where strengthened legislation on pollution coming from sanitation systems leads to conservation and sustainable use of oceans and their ecosystems	- Kimball LA. *International Ocean Governance: using international law and organizations to manage marine resources sustainably*. Revised ed. IUCN – The World Conservation Union. Gland: International Union for Conservation of Nature and Natural Resources (IUCN); 2003.
**Goal 15. Protect, restore and promote sustainable use of terrestrial ecosystems, sustainably manage forests, combat desertification, and halt and reverse land degradation and halt biodiversity loss**								
**15.1**	By 2020, ensure the conservation, restoration and sustainable use of terrestrial and inland freshwater ecosystems and their services, in particular forests, wetlands, mountains and drylands, in line with obligations under international agreements	**1**	**1**	**1**			(A) Target requires conservation of ecosystems which includes pollution reduction and safe disposal of human waste for both ecological purposes (B) Multiple synergies identified, for example, where improvements of treatment, re-use and disposal sanitation systems lead to reduced pollution; or with the sustainable use of ecosystems to treat sewage (e.g. green infrastructure, reed beds)	- UN-Water. Towards a worldwide assessment of freshwater quality: a UN-water analytical brief. UN-Water Analytical Brief. Geneva; 2016. - Simha P, Ganesapillai M. Ecological sanitation and nutrient recovery from human urine: how far have we come? A review. *Sustain Environ. Res.* 2017;27:107–16. - Thevenon F. Sustainable sanitation systems: health, environment and governance challenges. Geneva: WaterLex; 2017. - Metcalfe CD, Nagabhatla N, Fitzgerald SK. Multifunctional wetlands: pollution abatement by natural and constructed wetlands. In: *Multifunctional Wetlands*. Cham: Springer; 2018. p. 1–14. - Koottatep T, Surinkul N, Polprasert C, Kamal ASM, Koné D, Montangero A, et al. Treatment of septage in constructed wetlands in tropical climate: lessons learnt from seven years of operation. *Water Sci Technol.* 2005;9:119–26. - Jordan MJ, Nadelhoffer KJ, Fry B. Nitrogen cycling in forest and grass ecosystems irrigated with 15N-enriched wastewater. *Ecol Appl.* 1997;3:864–81.
**15.2**	By 2020, promote the implementation of sustainable management of all types of forests, halt deforestation, restore degraded forests and substantially increase afforestation and reforestation globally		**1**	**1**			(B) Multiple synergies, for example where sewage sludge application on soils of forests leads to increase in nutrients which benefit ecosystems; where forest covers helps reduce sanitation risks; and where biogas development reduces need for wood; also where development of sanitation systems leads to deforestation	- Bramryd T. Impact of sewage sludge application on the long-term nutrient balance in acid soils of scots pine (Pinus Sylvestris, L.) forests. *Water Air Soil Pollut.* 2002;140:381–99. - Bramryd T. Long-term effects of sewage sludge application on the heavy metal concentrations in acid pine (Pinus sylvestris L.) forests in a climatic gradient in Sweden. *For Ecol Manage.* 2013;289:434–44. - Zasoski R, Edmonds R, Bledsoe C, Henry C, Vogt D, Vogt K, et al. Municipal sewage sludge use in forests of the pacific northwest, U.S.A.: environmental concerns. *Waste Manag Res.* 1984;2:227–46. - Harvey P. *Excreta disposal in emergencies: a field manual*. Loughborough: Loughborough University; 2007.
**15.3**	By 2030, combat desertification, restore degraded land and soil, including land affected by desertification, drought and floods, and strive to achieve a land degradation-neutral world		**1**	**1**			(B) Synergies, for example, where treated urine is re-used to irrigate plant cover to harnessing deserts and preventing desertification	- Glaser B. Prehistorically modified soils of central Amazonia: a model for sustainable agriculture in the twenty-first century. *Philos Trans R Soc B Biol Sci.* 2007;362:187–96. - Factura H, Bettendorf T, Buzie C, Pieplow H, Reckin J, Otterpohl R. Terra Preta sanitation: re-discovered from an ancient Amazonian civilisation – integrating sanitation, bio-waste management and agriculture. *Water Sci Technol.* 2010;61:2673–9.
**15.4**	By 2030, ensure the conservation of mountain ecosystems, including their biodiversity, in order to enhance their capacity to provide benefits that are essential for sustainable development	**1**	**1**	**1**			(A) Target calls for action where human waste represents a significant ecological issue to mountain ecosystems (B) Synergies, for example, where safe disposal of waste reduces pollution on mountain ecosystems or helps enhancing them	- Apollo M. The good, the bad and the ugly – three approaches to management of human waste in a high-mountain environment. *Int J Environ Stud.* 2017;74:129–58. - Boehler M, Joss A, Buetzer S, Holzapfel M, Mooser H, Siegrist H. Treatment of toilet wastewater for reuse in a membrane bioreactor. *Water Sci Technol.* 2007;56:63–70.
**15.5**	Take urgent and significant action to reduce the degradation of natural habitats, halt the loss of biodiversity and, by 2020, protect and prevent the extinction of threatened species	**1**	**1**	**1**			(A) Target requires action for biodiversity protection that occurs with controlled disposal of human waste and threatens species and their habitats (e.g. excess in nutrients leading to eutrophication, activation of pharmaceutical, etc.) (B) Evidence of natural habitats and biodiversity enhanced with re-use of faeces	- European Commission. The factory of life: why soil biodiversity is so important. Luxembourg, European Commission; 2010.
**15.6**	Promote fair and equitable sharing of the benefits arising from the utilisation of genetic resources and promote appropriate access to such resources, as internationally agreed							
**15.7**	Take urgent action to end poaching and trafficking of protected species of flora and fauna and address both demand and supply of illegal wildlife products							
**15.8**	By 2020, introduce measures to prevent the introduction and significantly reduce the impact of invasive alien species on land and water ecosystems and control or eradicate the priority species		**1**		**1**		(B) Synergies may occur where sustainable sanitation systems reduce pollution and reinforce ecosystem equilibrium for native plant species to thrive (C) Trade-offs could occur as non-native species may be introduced with certain types of sanitation systems (e.g. prioritisation of introduced plants that are cultivated with human excreta)	
**15.9**	By 2020, integrate ecosystem and biodiversity values into national and local planning, development processes, poverty reduction strategies and accounts		**1**	**1**			(B) Synergies include planning strategies that integrate ecosystem and biodiversity development through re-use and safe disposal of human waste	- Bryars CK. *Planning [and] the sanitary city: understanding implications of community-based ecological sanitation reforms in the U.S.* University of Massachusetts Amherst; 2016.
**15.a**	Mobilise and significantly increase financial resources from all sources to conserve and sustainably use biodiversity and ecosystems		**1**	**1**			(B) Synergies include financial investment in the conservation and sustainable use of ecosystems through re-use of and safe disposal of human waste	- Werner C, Panesar A, Rüd SB, Olt CU. Ecological sanitation: principles, technologies and project examples for sustainable wastewater and excreta management. *Desalination.* 2009;248:392–401.
**15.b**	Mobilise significant resources from all sources and at all levels to finance sustainable forest management and provide adequate incentives to developing countries to advance such management, including for conservation and reforestation		**1**	**1**			(B) Synergies as per Target 15.2	
**15.c**	Enhance global support for efforts to combat poaching and trafficking of protected species, including by increasing the capacity of local communities to pursue sustainable livelihood opportunities							
**Goal 16. Promote peaceful and inclusive societies for sustainable development, provide access to justice for all and build effective, accountable and inclusive institutions at all levels**								
**16.1**	Significantly reduce all forms of violence and related death rates everywhere	**1**	**1**	**1**			(A) Target calls for action in sanitation, including where facilities are places of violence (B) Multiple synergies, including where safer facilities reduce violence towards girls and women	- Corburn J, Hildebrand C. Slum sanitation and the social determinants of women’s health in Nairobi, Kenya. *J Environ Public Health.* 2015;2015:1–6. - Belur J, Parikh P, Daruwalla N, Joshi R, Fernandes R. Perceptions of gender-based violence around public toilets in Mumbai slums. *Int J Comp Appl Crim Justice*. 2017;1–2:63–78.
**16.2**	End abuse, exploitation, trafficking and all forms of violence against and torture of children							
**16.3**	Promote the rule of law at the national and international levels and ensure equal access to justice for all							
**16.4**	By 2030, significantly reduce illicit financial and arms flows, strengthen the recovery and return of stolen assets and combat all forms of organised crime						
**16.5**	Substantially reduce corruption and bribery in all their forms	**1**	**1**	**1**			(A) Target calls for action in sanitation, for example, where corruption impacts access to sanitation (B) Synergies, for example, where reduced corruption increased sanitation access	- Jenkins M. The impact of corruption on access to safe water and sanitation for people living in poverty. *Anti-Corrruption Res Cent*. 2017;6. - Khan S. *Swachh Bharat Mission (urban): needs vs planning*. New Delhi: Centre for Policy Research; 2018.
**16.6**	Develop effective, accountable and transparent institutions at all levels	**1**	**1**	**1**			(A) Target calls for action to increase accountability to create inclusive decision-making in sanitation (B) Synergies, for example, with strenghthened capacity of institutions and enabling environment leading to improved provision and management of the sanitation sector	- Kennedy-Walker R, Amezaga JM, Paterson CA. The role of power, politics and history in achieving sanitation service provision in informal urban environments: a case study of Lusaka, Zambia. *Environ Urban.* 2015;27:489–504. - Khan S. *Swachh Bharat Mission (urban): needs vs planning*. New Delhi: Centre for Policy Research; 2018.
**16.7**	Ensure responsive, inclusive, participatory and representative decision-making at all levels	**1**	**1**	**1**			(A) Target calls for action to increase accountability to create inclusive decision-making in sanitation (B) Synergies include inclusive governance in sanitation that leads to improved access	- Seppälä OT. Effective water and sanitation policy reform implementation: need for systemic approach and stakeholder participation. *Water Policy.* 2002;4:367–88. - Khan S. *Swachh Bharat Mission (urban): needs vs planning*. New Delhi: Centre for Policy Research; 2018.
**16.8**	Broaden and strengthen the participation of developing countries in the institutions of global governance							
**16.9**	By 2030, provide legal identity for all, including birth registration							
**16.10**	Ensure public access to information and protect fundamental freedoms, in accordance with national legislation and international agreements		**1**	**1**			(B) Synergies, for example, where better freedom of choices and information enables enhanced sanitation access	- Miletzki J, Broten N. *Development as freedom*. London: Macat International; 2017.
**16.a**	Strengthen relevant national institutions, including through international cooperation, for building capacity at all levels, in particular in developing countries, to prevent violence and combat terrorism and crime		**`**					
**16.b**	Promote and enforce non-discriminatory laws and policies for sustainable development	**1**	**1**	**1**			(A) Call for action to ensure inclusive laws and policies in relation to sanitation (B) Multiple synergies including in the pursuit of justice for all which involves access to sanitation by all	- House S, Ferron S, Cavill S. *Scoping and diagnosis of the global sanitation fund’s approach to Equality and Non-Discrimination (EQND)*. GSF and WSSCC; 2017.
**Goal 17. Strengthen the means of implementation and revitalise the global partnership for sustainable development**								
**17.1**	Strengthen domestic resource mobilisation, including through international support to developing countries, to improve domestic capacity for tax and other revenue collection		**1**	**1**	**1**	**1**	(B) Evidence that improved domestic resource mobilisation can improve sanitation systems; and that sanitation sytems may support tax and revenue collection at national level particularly where willingness to pay for such services is high (C) But also trade-offs if taxes add burden on households which are prevented from investing in sanitation; or where national revenues from taxes do not go into sanitation	- Bisaga I, Norman G, Drabble S. *How can we influence municipal governments to allocate more money to sanitation?* London: Water & Sanitation for the Urban Poor; 2015. - UNICEF. Water and sanitation budget brief. UNICEF; 2016.
**17.2**	Developed countries to implement fully their official development assistance commitments, including the commitment by many developed countries to achieve the target of 0.7% of ODA/GNI to developing countries and 0.15–0.20% of ODA/GNI to least developed countries; ODA providers are encouraged to consider setting a target to provide at least 0.20% of ODA/GNI to least developed countries		**1**	**1**			(B) Synergies exist where commitments are targeted towards sanitation. Evidence shows that countries with the least coverage persistently received far less ODA per capita than did countries with much more extensive water and sanitation coverage, suggesting that ODA for water and sanitation is poorly targeted	- Cha S, Mankadi PM, Elhag MS, Lee Y, Jin Y. Trends of improved water and sanitation coverage around the globe between 1990 and 2010: inequality among countries and performance of official development assistance. *Glob Health Action.* 2017;10:1327170. - Newborne P, Tucker J, Bayliss K. *Strengthening pro-poor targeting of investments by African utilities in urban water and sanitation – the role of the International Development Association of the World Bank*. London: Water Aid; 2012.
**17.3**	Mobilise additional financial resources for developing countries from multiple sources		**1**	**1**			(B) Synergies where blended finance strengthens sanitation systems	- OECD. Meeting the challenge of financing water and sanitation. Paris: OECD; 2011. - World Water Council. Increasing financial flows for urban sanitation. Marseille: World Water Council; 2018. - Goksu A, Trémolet S, Kolker J, Kingdom B. Easing the transition to commercial finance for sustainable water and sanitation. Washington DC: The World Bank; 2017.
**17.4**	Assist developing countries in attaining long-term debt sustainability through coordinated policies aimed at fostering debt financing, debt relief and debt restructuring, as appropriate, and address the external debt of highly indebted poor countries to reduce debt distress		**1**	**1**	**1**	**1**	(B) Synergies where policies foster debt financing, relief and/or restructuring and enable investment in sanitation (C) Potential trade-offs where debt still poses major barriers to the sanitation sector or where sanitation increases accumulation of further debts	- Newborne P, Tucker J, Bayliss K. *Strengthening pro-poor targeting of investments by African utilities in urban water and sanitation – the role of the International Development Association of the World Bank.* London: Water Aid; 2012. - Annamraju S, Calaguas B, Gutierrez E. *Financing water and sanitation: key issues in increasing resources to the sector*. Water Aid briefing paper. London: WaterAid; 2001.
**17.5**	Adopt and implement investment promotion regimes for least developed countries		**1**	**1**			(B) Synergies where such investment promotion regimes benefit the sanitation sector	- Gopalan S, Rajan RS. Has Foreign aid been effective in the water supply and sanitation sector? Evidence from panel data. *World Dev.* 2016;85:84–104. - Khan S. *Swachh Bharat Mission (urban): needs vs planning*. New Delhi: Centre for Policy Research; 2018.
**17.6**	Enhance North-South, South-South and triangular regional and international cooperation on and access to science, technology and innovation and enhance knowledge sharing on mutually agreed terms, including through improved coordination among existing mechanisms, in particular at the United Nations level, and through a global technology facilitation mechanism		**1**	**1**			(B) Multiple synergies exist as a result of international cooperation and knowledge sharing leading to increased sanitation access	- Murphy HM, McBean EA, Farahbakhsh K. Appropriate technology – a comprehensive approach for water and sanitation in the developing world. *Technol Soc.* 2009;31:158–67.
**17.7**	Promote the development, transfer, dissemination and diffusion of environmentally sound technologies to developing countries on favourable terms, including on concessional and preferential terms, as mutually agreed		**1**	**1**			(B) Synergies as per Target 17.6	
**17.8**	Fully operationalise the technology bank and science, technology and innovation capacity-building mechanism for least developed countries by 2017 and enhance the use of enabling technology, in particular information and communications technology		**1**	**1**			(B) Synergies as per Target 17.6	- Glöckner H, Mkanga M, Ndezi T. Local empowerment through community mapping for water and sanitation in Dar es Salaam. *Environ Urban.* 2004;16:185–98. - McGranahan G, Walnycki A, Dominick F, Willard K, Kyessi A, Mtwangi Limbumba T, et al. *Universalising water and sanitation coverage in urban areas: from global targets to local realities in Dar es Salaam, and back*. London: IIED; 2016.
**17.9**	Enhance international support for implementing effective and targeted capacity-building in developing countries to support national plans to implement all the sustainable development goals, including through North-South, South-South and triangular cooperation		**1**	**1**			(B) Synergies as per Target 17.6	- As per Target 17.6
**17.10**	Promote a universal, rules-based, open, non-discriminatory and equitable multilateral trading system under the World Trade Organization, including through the conclusion of negotiations under its Doha Development Agenda							
**17.11**	Significantly increase the exports of developing countries, in particular with a view to doubling the least developed countries’ share of global exports by 2020							
**17.12**	Realise timely implementation of duty-free and quota-free market access on a lasting basis for all least developed countries, consistent with World Trade Organization decisions, including by ensuring that preferential rules of origin applicable to imports from least developed countries are transparent and simple, and contribute to facilitating market access							
**17.13**	Enhance global macroeconomic stability, including through policy coordination and policy coherence							
**17.14**	Enhance policy coherence for sustainable development	**1**	**1**	**1**			(A) Target calls for action as sustainable development cannot be achieved without access to sanitation (B) Synergies where this coincides with the sustainable development of sanitation systems	- UN Environment. Progress on integrated water resources management. Global Baseline for SDG 6 Indicator 6.5.1: degree of IWRM Implementation. 2018. - Newborne P, Ranaivoari A, Rabeantoandro F. Sanitation and hygiene in developing countries: identifying and responding to barriers. 2007.
**17.15**	Respect each country’s policy space and leadership to establish and implement policies for poverty eradication and sustainable development		**1**	**1**			(B) Synergies where these policies for sustainable development (as per Target 17.14) and poverty eradiction are associated to investments in adequate, equitable and dignified sanitation	- OECD. Partnerships to enhance policy coherence for sustainable development. In: *Policy Coherence For Sustainable Development 2017: eradicating poverty and promoting prosperity*. Paris: OECD Publishing; 2017. - Newborne P, Ranaivoari A, Rabeantoandro F. Sanitation and hygiene in developing countries: identifying and responding to barriers. 2007.
**17.16**	Enhance the global partnership for sustainable development, complemented by multi-stakeholder partnerships that mobilise and share knowledge, expertise, technology and financial resources, to support the achievement of the sustainable development goals in all countries, in particular developing countries		**1**	**1**			(B) Multiple synergies as multi-stakeholder partnerships can help foster better access to sanitation	- ODI, FDC. Multi-Stakeholder Partnerships Issue Paper Pulling together to uplift and empower the world. Kuala Lumpur: GKP; 2003. - Murphy HM, McBean EA, Farahbakhsh K. Appropriate technology – a comprehensive approach for water and sanitation in the developing world. *Technol Soc.* 2009;31:158–67.
**17.17**	Encourage and promote effective public, public-private and civil society partnerships, building on the experience and resourcing strategies of partnerships		**1**	**1**			(B) Synergies as per Target 17.16	- Allen A, Hofmann P, Mukherjee J, Walnycki A. Water trajectories through non-networked infrastructure: insights from peri-urban Dar es Salaam, Cochabamba and Kolkata. *Urban Res Pract.* 2017;10:22–42.
**17.18**	By 2020, enhance capacity-building support to developing countries, including for least developed countries and Small Island Developing States, to increase significantly the availability of high-quality, timely and reliable data disaggregated by income, gender, age, race, ethnicity, migratory status, disability, geographic location and other characteristics relevant in national contexts		**1**	**1**			(B) Multiple synergies, for example, where capacity-building to developing countries through or trainings in the sanitation sector lead to improved access to sanitation	- McGranahan G, Walnycki A, Dominick F, Willard K, Kyessi A, Mtwangi Limbumba T, et al. *Universalising water and sanitation coverage in urban areas: from global targets to local realities in Dar es Salaam, and back*. London: IIED; 2016. - Cotton A, Bartram J. Sanitation: On- or off-track? Issues of monitoring sanitation and the role of the joint monitoring programme. *Waterlines.* 2008;27:12–29.
**17.19**	By 2030, build on existing initiatives to develop measurements of progress on sustainable development that complement gross domestic product, and support statistical capacity-building in developing countries		**1**	**1**			(B) Multiple synergies that include capacity-building leading to progress tracking and action-taking from strong Monitoring and Evaluation (M&E) processes, and supporting the identification of gaps where action in sanitation is needed	- Cotton A, Bartram J. Sanitation: On- or off-track? Issues of monitoring sanitation and the role of the joint monitoring programme. *Waterlines.* 2008;27:12–29.
	**COUNT**	**83**	**131**	**130**	**29**	**28**
